# DIRProt: a computational approach for discriminating insecticide resistant proteins from non-resistant proteins

**DOI:** 10.1186/s12859-017-1587-y

**Published:** 2017-03-24

**Authors:** Prabina Kumar Meher, Tanmaya Kumar Sahu, Anjali Banchariya, Atmakuri Ramakrishna Rao

**Affiliations:** 1 Division of Statistical Genetics, ICAR-Indian Agricultural Statistics Research Institute, New Delhi, 110012 India; 2 Centre for Agricultural Bioinformatics, ICAR-Indian Agricultural Statistics Research Institute, New Delhi, 110012 India; 3 Department of Bioinformatics, Janta Vedic College, Baraut, Baghpat, 250611 Uttar Pradesh India

**Keywords:** Insecticide resistance, SVM, Di-peptide composition, Cytochrome P450, GABA

## Abstract

**Background:**

Insecticide resistance is a major challenge for the control program of insect pests in the fields of crop protection, human and animal health etc. Resistance to different insecticides is conferred by the proteins encoded from certain class of genes of the insects. To distinguish the insecticide resistant proteins from non-resistant proteins, no computational tool is available till date. Thus, development of such a computational tool will be helpful in predicting the insecticide resistant proteins, which can be targeted for developing appropriate insecticides.

**Results:**

Five different sets of feature viz., amino acid composition (AAC), di-peptide composition (DPC), pseudo amino acid composition (PAAC), composition-transition-distribution (CTD) and auto-correlation function (ACF) were used to map the protein sequences into numeric feature vectors. The encoded numeric vectors were then used as input in support vector machine (SVM) for classification of insecticide resistant and non-resistant proteins. Higher accuracies were obtained under RBF kernel than that of other kernels. Further, accuracies were observed to be higher for DPC feature set as compared to others. The proposed approach achieved an overall accuracy of >90% in discriminating resistant from non-resistant proteins. Further, the two classes of resistant proteins i.e., detoxification-based and target-based were discriminated from non-resistant proteins with >95% accuracy. Besides, >95% accuracy was also observed for discrimination of proteins involved in detoxification- and target-based resistance mechanisms. The proposed approach not only outperformed Blastp, PSI-Blast and Delta-Blast algorithms, but also achieved >92% accuracy while assessed using an independent dataset of 75 insecticide resistant proteins.

**Conclusions:**

This paper presents the first computational approach for discriminating the insecticide resistant proteins from non-resistant proteins. Based on the proposed approach, an online prediction server DIRProt has also been developed for computational prediction of insecticide resistant proteins, which is accessible at http://cabgrid.res.in:8080/dirprot/. The proposed approach is believed to supplement the efforts needed to develop dynamic insecticides in wet-lab by targeting the insecticide resistant proteins.

**Electronic supplementary material:**

The online version of this article (doi:10.1186/s12859-017-1587-y) contains supplementary material, which is available to authorized users.

## Background

Insecticides are used to control the insects affecting the agricultural crops, parasitizing livestock, as well as to eradicate the pests transmitting dangerous infectious diseases. However, frequent application of insecticides has resulted in the resurgence of pests and appearance of resistant pest species. Insecticide resistance is the heritable change in the sensitivity of a pest population that is reflected in the repeated failure of a product (insecticides) to achieve the expected level of control when used according to the level of recommendation for that pest species [1]. Several studies have indicated the involvement of multiple genes in conferring the resistance to many insect species [2–4]. Thus, characterization of these genes is useful to understand the development of resistance and designing new strategies to minimize the development of insecticide resistance [5].

Three major mechanisms are involved in insecticide resistance [5]: (i) detoxification of insecticides through alteration in the activities of enzymes like esterase, oxidases or glutathione S-transferases (GSTs) that prevents the insecticide from reaching to its site of action [6–8], (ii) Insensitivity of the insecticide target proteins [9, 10], because of which the insecticide no longer binds to its target [11, 12] and (iii) reduction in insecticide uptake due to decrease in permeability of insect cuticle [13, 14]. Though there is evidence of alteration in cuticular penetration, most of the studies have focused and evaluated the target site insensitivity and detoxification of insecticides (metabolic resistance) mechanisms. Moreover, these two mechanisms have been reported to cover a wide range of resistance levels to almost all available insecticides [9].

The cytochrome P450 family of genes in insect play an important role in the detoxification of insecticides resulted in the development of resistance to insecticides [4, 15, 16]. Besides, GSTs have also been reported to be involved in the detoxification of insecticides [17, 18]. As far as target-based mechanism is concerned, there are three main targets for conventional insecticides viz., GABA (γ-amino butyric acid)-gated chloride ion channel, voltage-gated ion channel and acetylcholinesterases [19]. The GABA receptor is the site of target for cyclodiene (dieldrin) insecticides [20], where the resistance to dieldrin (*Rdl*) is conferred by the change of a single amino acid in GABA-gated chloride ion channel encoded by *Rdl* gene [21]. Further, knockdown resistance (*Kdr*) is one of the major forms of resistance to DDT and pyrethroid insecticides [22], which is associated with mutations in the voltage-gated sodium channel [22–25]. Acetylcholinesterase (AChE) in nerve synapses is the target protein for the insecticides like organophosphorus (e.g., malathion, fenitrothion) and carbamate (e.g., propoxur, sevin) [12]. The point mutation in the insecticide-binding site of AChE has been identified as the cause of insensitivity to these insecticides [26].

The above mentioned works help enable to understand the molecular mechanisms involved in the insecticide resistance. Further, the analysis of bio-molecules involved in this phenomenon has confirmed the importance of single genes in target site resistance and involvement of multi-gene families like cytochrome P450 in metabolic resistance [27]. Several studies on the effects of mutational changes in target proteins on insecticide resistance aid to the knowledge on the insect proteins involved in this process. For instance, Riveron et al. [28] demonstrated that the single amino acid change (L119F) in an up regulated GST gene, GSTe2, confers high level of metabolic resistance to DDT in the malaria vector *Anopheles funestus*. In another study, Nwane et al. [29] identified that two mutations at position 1014 of the S_6_ transmembrane segment of domain II in the voltage gated sodium channel i.e., leucine to a phenylalanine (L1014F) or to a serine (L1014S) confers resistance to DDT and pyrethroid insecticides in *Anopheles gambiae*. In the recent past, several studies have identified species-specific insecticide resistant genes through transcriptome and expression profile analysis. Hsu et al. [30] identified 90 P450, 42 GST, 31 CoE-related genes in *Bactrocera doralis*, representing three major enzyme families involved in insecticide metabolism and resistance. In another study, 49 P450, 31 GST and 21 CES-specific genes of *Liposcelis bostrychophila* were reported to be involved in insecticide resistance, through transcriptome and differential gene expression analysis [31]. Recently, Cui et al. [32] identified relevant genes in response to flubendiamide insecticide in Asian corn borer (*Ostrinia furnacalis*), through *de novo* transcriptome and expression-profile analysis.

Though the transcriptome and expression profile analysis is one way of identifying the resistance genes, it is species specific. Moreover the expression profile analysis is expensive as well as time consuming. Thus, development of a computational tool for identifying the resistant genes independent of the species and economically as well would help in augmenting the research related to the identification of insecticide resistant genes. However, no computational tool is reported till date for the discrimination of insecticide resistant proteins from the proteins that do not confer resistance. Keeping this in view, we propose a computational approach to discriminate the insecticide resistant proteins from non-resistant proteins. The developed computational approach can be used for identification of the resistant proteins across species as well as with minimum resource (cost and time). We have also developed an online prediction server that can be easily used by experimental scientist and researchers to predict an unknown protein sequence as either insecticide-resistant or non-resistant protein. Moreover, computational identification of insecticide resistant proteins will supplement the efforts needed to develop insecticides in targeting the resistance proteins.

## Methods

### Collection and processing of data

In this study, protein sequences corresponding to four important groups of insecticide resistant genes viz., cytochrome P450, Kdr, Rdl and AChE were collected from insecticide resistance gene database (http://www.cib.res.in/irgd/). We considered these four categories of genes because they represent important families of insecticide resistant genes which are resistant to commonly used insecticides. Besides, the resistant protein sequences were reported to be involved in two important resistance mechanisms viz., detoxification-based and target-based. Further, target-based resistant proteins are confined to three main targets of insecticides i.e., AChE, GABA-gated chloride ion channel and voltage-gated sodium channel. A total of 822 sequences (772 cytochrome P450, 30 AChE, 17 Rdl and 3 Kdr) belonging to 11 insect species (Additional file 1) were collected. Initially, we removed the sequences having non-standard residues. Then, four positive sets having 128, 285, 349 and 442 sequences were prepared, where the maximum pair-wise sequence identities were 40%, 60%, 70% and 90% respectively. The sequences with more than considered level of pair-wise sequence identity were removed using CDHIT [33]. For negative set, protein sequences (other than the positive sets) of the considered species were collected from the Uniprot (http://www.uniprot.org/) database. For the species *Acyrthosiphon pisum* and *Tribolium castaneum*, only the reviewed sequences were collected, as large number of sequence are present in Uniprot for these two species. On the other hand, all the sequences available for remaining nine species were collected. After removing the sequences having non-standard residues as well as the identical sequences, a total of 12613 sequences were obtained. Further, to avoid homologous bias in the negative dataset, sequences with >40% pair-wise identity were removed using CDHIT. Finally a dataset with 3919 sequences was obtained and considered as the negative dataset.

### Feature generation

Protein sequences are the strings of amino acid residues, and hence they need to be mapped onto numeric feature vectors before being used as input in machine learning classifier. In this study, amino acid composition (AAC), di-peptide composition (DPC), pseudo amino acid composition (PAAC), composition-transition-distribution (CTD) and auto correlation function (ACF) were used to transform the protein sequences into numeric feature vectors.

#### Amino acid composition (AAC)

AAC is a basic feature of protein sequence [34], which is closely associated with its attributes, such as sub-cellular location [35, 36], secondary structure content [37] and domain [38]. AAC consists of 20 discrete numbers, each of which represents the frequency of the native amino acids in a protein sequence. Based on the AAC, each protein sequence was encoded into a 20-dimensional numerical vector.

#### Di-peptide composition (DPC)

One of the limitations of AAC is that it does not take into account the local order information of amino acids in the protein. On the other hand, DPC, which gives a fixed pattern length of 400 (20 × 20), encapsulates the global information about each protein sequence and the order it contains [39]. For any di-peptide, its composition was computed as the ratio of the frequency of that di-peptide to the total number possible di-peptide in the protein sequence.

#### Pseudo amino acid composition (PAAC)

The concept of PAAC was originally introduced by Chou [40] for predicting the protein sub-cellular locations and membrane protein types. Based on the conventional AAC, Chou proposed a set of discrete numbers to take into account the sequence order effects. PAAC has been proven to be an extremely effective feature in many proteins and protein-related systems [41]. The PAAC for each protein sequence can be represented by a (20 + d)-dimensional vector for d-tier correlation factor. Here, the PAAC was extracted for 1^st^-tier correlation only, by which each sequence was transformed into a 21-dimensional numeric vector. For further details, one can refer to [40, 42, 43].

#### Composition-transition-distribution (CTD)

The CTD feature was introduced by Dubchak et al. [44] for predicting protein folding classes. Thereafter, the CTD feature has been adopted by many researchers for protein function and structure studies [45, 46]. In CTD feature, composition (C) is the number of amino acids of a particular type divided by the total number of amino acids. Transition (T) characterizes the frequency percentage with which amino acids of a particular type is followed by other amino acids. Distribution (D) measures the chain length within which the first 25%, 50%, 75% and 100% of the amino acids of a particular type is located respectively. Based on the CTD feature, each protein sequence of length *L* was encoded to a L+{L*(L-1)/2} + (L*5)-dimension numeric vector.

#### Auto correlation function (ACF)

Sequence autocorrelation-based features assume that the disturbances in each area are systematically related to those in adjacent areas [47]. This concept helps to analyze the dependency among the features of sequences in each location. Autocorrelation features were computed based on the distribution of amino acid properties along the sequence, using all the 531 amino acid indices available in AAindex database [48]. In this feature encoding, for an autocorrelation of order *n*, each sequence was transformed into a numeric vector of length 531*n.

### Supervised learning technique

For classification purpose we used the support vector machine (SVM), which is a nonparametric algorithm developed by Vapnik [49]. It is a very promising and popular method for pattern recognition that has been widely used for prediction purpose in the field of bioinformatics [50–56]. It is proven to be very efficient in many biological analyses due to their ability to handle noise and large input dataset [57, 58]. A brief description about the working principle of SVM is described as follows:

Consider a binary classification problem with *N* samples or input vectors **x**_*i*_ ∈ *R* ^*d*^, (*i* = 1, 2, …, *N*), where **x**_*i*_ with class levels *y*_*i*_ ∈ {−1, 1} can be considered as the *i* ^th^ protein or vector defined in *d*-dimensional space (which depends upon the sequence encoding approach). In present work, 1 refers to resistant class and −1 represents non-resistant class. The objective here is to construct a binary classifier from the available sample (training set) that has less probability of misclassifying future sample (test set). Non-linear SVM maps input vectors **x**_*i*_ ' *s* into high dimensional feature space and constructs an optimal separating hyper-plane (OSH) that maximizes the distance between hyper-plane and nearest data points of each class in the space. Mathematically, the hyper-plane is represented as *y* = sgn(**w** ^*T*^ **x** + *b*), where **w** represents a weight vector that can map training data in the input space to the outer space and *b* represents bias. For a two class problem, it can be formulated as

$$ \left\{\begin{array}{l}{\mathbf{w}}^T{\mathbf{x}}_i+ b\ge 1\kern0.48em  if\kern0.24em {y}_i=1\\ {}{\mathbf{w}}^T{\mathbf{x}}_i+ b\le -1\kern0.48em  if\kern0.24em {y}_i=-1\end{array}\right.. $$

The SVM training procedure involves optimization of convex quadratic problem i.e., with lagrangian multipliers *α*_*i*_ ≥ 0, $\mathrm{maximize}\kern0.24em {\displaystyle \sum_{i=1}^N{\alpha}_i-\frac{1}{2}{\displaystyle \sum_{i=1}^N{\displaystyle \sum_{j=1}^N{\alpha}_i{\alpha}_j{y}_i{y}_j K\left({\mathbf{x}}_i{\mathbf{x}}_j\right)}}}$ subject to the constraints 0 ≤ *α*_*i*_ ≤ *c* (*i* = 1, 2, …, *N*) and ${\displaystyle \sum_{i=1}^N{\alpha}_i{y}_i=0}$, where *c* is the regularization parameter that controls trade-off between margin and classification error. The **x**_*j*_ ' *s* are called support vectors only if corresponding *α*_*j*_ > 0. After the SVM has been trained, the decision function for classification of query sequence (**x**) can be formulated as

$$ f\left(\mathbf{x}\right)=\operatorname{sgn}\left({\displaystyle \sum_{i=1}^N{y}_i{\alpha}_i K\left(\mathbf{x}.{\mathbf{x}}_i\right)+ b}\right) $$.

The choice of the proper kernel function *K* is important to train SVM model because the power of SVM comes from the kernel representation that allows the nonlinear mapping of input space to a higher dimensional feature space. In this work, four commonly used kernel functions [59] viz., linear (**x**_*i*_^′^ **x**_*j*_), polynomial ((*γ* **x**_*i*_^′^ **x**_*j*_ + *r*)^*d*^), radial basis (− exp{−*γ*‖**x**_*i*_ − **x**_*j*_‖^2^}) and sigmoid (tanh(*γ* **x**_*i*_^′^ **x**_*j*_ + *r*)) were used, where *r*, *d*, *γ* >0 are the kernel parameters.

### Validation of the model

Cross-validation procedure has been widely accepted for assessing the performance of classifiers [60]. Thus, we used the 10-fold cross-validation to assess the performance of our approach. It was carried out by partitioning the dataset into 10 approximately equal-sized sets at random, where nine partitions were used to train the model and the remaining one part was used to assess the model accuracy. This process was repeated 10 times in such a way that each partition was tested once in the model.

### Performance evaluation

Different performance metrics viz., sensitivity (Sn), specificity (Sp), accuracy (Ac), precision (Pre) and Matthew’s correlation coefficient (MCC) were used to measure the accuracy of the developed prediction approach. The Sn, Sp, Ac, Pre and MCC parameters are defined as:*Sn* = *tp*/(*tp* + *fn*), *Sp* = *tn*/(*tn* + *fp*), *Ac* = (*tp* + *tn*)/(*tp* + *fn* + *tn* + *fp*), *Pre* = *tp*/(*tp* + *fp*), $M C C=\left[\left( tp\times tn\right)-\left( fp\times fn\right)\right]/\sqrt{\left( tp+ fn\right)\times \left( tp+ fp\right)\times \left( tn+ fn\right)\times \left( tn+ fp\right)}$. True positive (*tp*) is the number of resistant proteins correctly predicted as resistant proteins, true negative (*tn*) is the number of non-resistant proteins correctly predicted as non-resistant proteins, false negative (*fn*) is the number of resistant proteins incorrectly predicted as non-resistant proteins and false positive (*fp*) is the number of non- resistant proteins incorrectly predicted as resistant proteins. Besides the above mentioned performance metrics, area under receiving operating characteristic curve (AUC-ROC) [61] was also used to measure the predictive ability. For given false positive rate (α) and true positive rate (1-β) at different threshold values, the AUC-ROC was computed as ${\displaystyle \sum_i\left\{\left(1-{\beta}_i.\varDelta \alpha \right)+\left(1/2\right)\left[\varDelta \left(1-\beta \right).\varDelta \alpha \right]\right\}}$, where *Δ*(1 − *β*) = (1 − *β*_*i*_) − (1 − *β*_*i* − 1_), *Δα* = *α*_*i*_ − *α*_*i* − 1_ and *i* = 1,2, …, *m* (number of test instances) [62]. A subroutine in R programming language was written to compute the values of these performance metrics.

### Training and testing datasets

Using four positive sets and one negative set (mentioned under “collection and processing of data”), four datasets were prepared that consists of both positive and negative sequences. Here each dataset contains a different positive set and the same negative set (3919 negative sequences). All the four datasets are highly unbalanced as the number sequences present in one class (non-resistant class) is much larger than the other class (resistant class). To avoid biasness towards the non-resistant class (major class) while predicting using machine learning classifier like SVM, balanced datasets were prepared that consists of same number of sequences from both the classes, where the sequences of the major class were randomly drawn from the available sequences of the major class. For instance, first balanced dataset contains 128 positive and 128 negative sequences, where the 128 negative sequences were randomly drawn from 3919 negative sequences. As the generalized predictive ability cannot be assured based on a single dataset, 100 sample sets were prepared, where each sample set consists of same number of positive and negative instances. Further, in each sample set, a 10-fold cross validation procedure was adopted. The performance metrics were computed by taking average over the 10 folds as well as over 100 sample sets.

### Mechanism-based classification

The insecticide resistance mechanism can be broadly categorized into two types, viz., target-based mechanism and detoxification-based mechanism. The Rdl, Kdr and AChE genes come under target-based and cytochrome P450 genes come under detoxification-based mechanism. To test whether the genes under these two categories are different or not, a binary classification was carried out by employing SVM, where 15 sequences (with <90% pair-wise sequence identity) from target-based and 452 sequences (with <90% pair-wise sequence identity) from detoxification-based category were used. Similar to the classification of resistant and non-resistant proteins, 100 sample sets were prepared where each sample set consists of 15 sequences from each class. Since, there are 452 sequences in the detoxification-based category, 15 sequences were randomly drawn each time. As the number of sequences in each sample is not large, leave-one-out cross validation (LOOCV) technique was adopted for classification of detoxification- and target-based resistant proteins. Here, detoxification-based category was considered as positive class and target-based category as negative class.

### Comparison with blast algorithm

Performance of the proposed approach was also compared with that of Blastp [63], PSI-Blast [64] and Delta-Blast [65], which are powerful algorithms to detect protein homologs. Further, comparison was made through 10-fold cross validation technique. For cross validation, offline (local) Blast software was used with *blastp*, *psiblast* and *deltablast* modules/programs in which the training set for each fold of cross validation was defined as the database and sequences of the corresponding test set were used as query. Each query sequence was predicted as the resistant or non-resistant category based on the top hit found in the blast search. Three different e-values i.e., 0.1, 1 and 10 were used to assess the performance of the Blastp, PSI-Blast and Delta-Blast. Furthermore, performance of the proposed approach was compared based on best feature set with which higher accuracies were obtained as compared to the other feature sets.

### Performance evaluation using independent dataset

To assess the generalized predictive ability of the proposed approach, its performance was further tested using an independent test dataset. The independent dataset was collected based on published literature that includes 53 cytochrome P450, 2 Kdr, 3 Rdl and 17 AChE proteins. Specifically, 115 cytochrome P450 genes were reported by Hsu et al. [30]. Out of 115, we used 53 as they are available in NCBI. Similarly, 2 Kdr, 3 Rdl and 17 AChE genes were collected from NCBI, based on the study of Zuo et al. [66], Wondji et al. [67] and Li and Han [68] respectively. Sequences of the independent test set are provided in Additional file 1.

### Development of prediction server

An online prediction server was developed using HTML and PHP, where the combination of best feature set and classifier was used. A developed R-code was executed in background upon submission of the sequences in FASTA format to the server. The user has to submit the protein sequences having only standard amino acid residues. This server can be used to predict the likelihood of any unknown protein sequence being an insecticide resistant protein with certain probability.

## Results

### Analysis of amino acid compositions

**Fig. 1:**
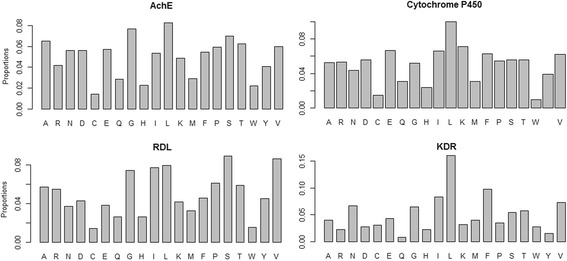
Composition of amino acids in all the four categories of insecticide resistant proteins. It is observed that proportions of leucine are higher, whereas proportions of cystene and tryptophan are lower in all the four categories

The composition of amino acids in four different groups of insecticide resistant proteins is shown in Fig. 1. It is seen that the proportions of leucine (L) are higher, whereas the proportions of cystene (C) and tryptophan (W) are lower in all the four categories.

### Analysis of kernel functions

**Fig. 2:**
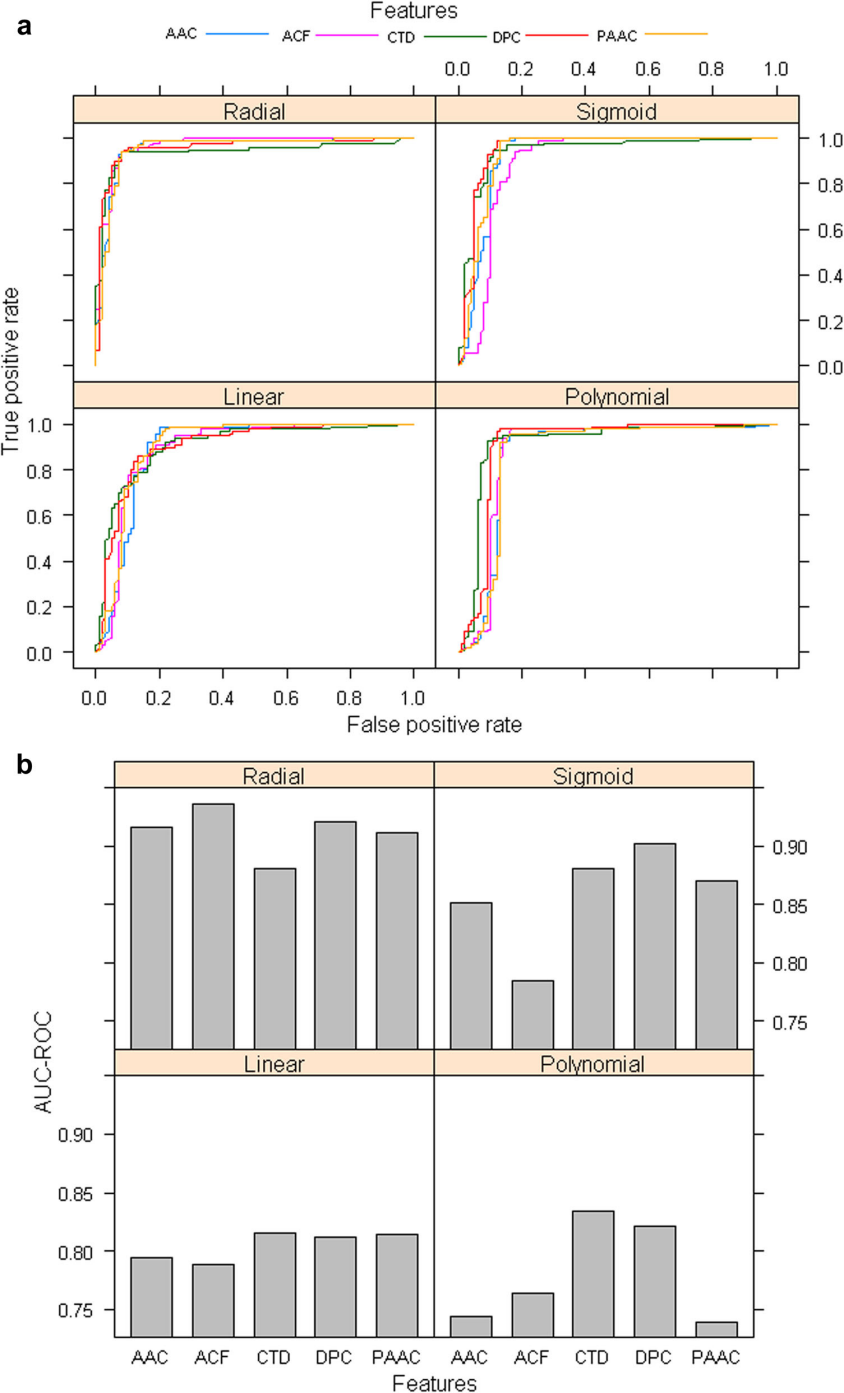
**a** ROC *curves* of SVM for different kernels and features, **b** bar plots of corresponding AUC-ROC values. It is seen that the AUC-ROC values are higher for RBF kernel as compared to other kernels

Based on a sample dataset consisting of 100 positive and 100 negative sequences that were drawn randomly from the available positive and negative sequences, performance of SVM was analyzed. ROC curves for all the four kernels as well as for all the five feature sets are shown in Fig. 2a and the corresponding AUC-ROC values are shown in bar plots (Fig. 2b). From the ROC curves it is not clear that which kernel is better, whereas from AUC-ROC plots it is clear that the values of AUC-ROC are higher for the RBF kernel, irrespective of the feature set used. Though in RBF kernel the AUC-ROC for ACF feature set is highest, it is difficult to choose the best feature set while other three kernels are taken into account. Therefore, all the feature sets and the RBF kernel were used for further analysis.

### Cross-validation performance analysis

**Fig. 3:**
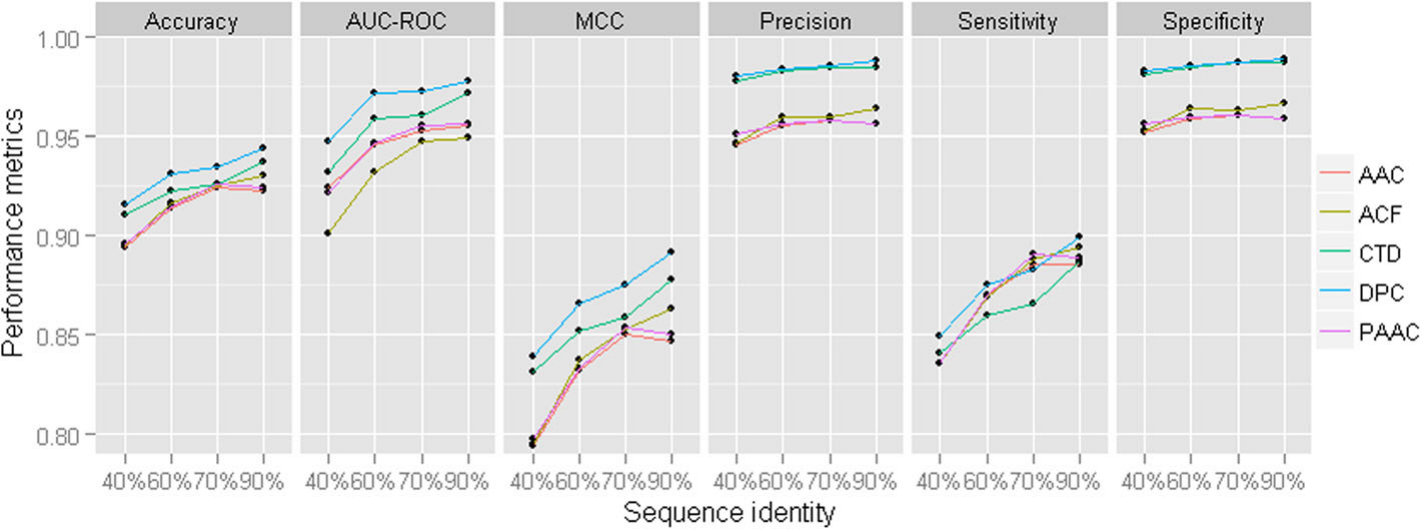
Performance metrics of SVM with RBF kernel for different feature sets and different percentage of pair-wise sequence identity in the positive set. It can be seen that the performance metrics are higher for DPC feature set as compared to other feature sets, irrespective of the percentage of sequence identity in the positive dataset

For all the four datasets (mentioned under “Training and testing datasets”) as well as for all the feature sets, performance metrics averaged over 10-fold as well as 100 sample sets are given in Table 1. Moreover, to analyze the trend in accuracies, performance metrics are also plotted in line graphs (Fig. 3). It is observed that the sensitivities are less as compared to the specificities (Table 1). Further, higher accuracies are observed for the dataset having resistant proteins with <90% pair-wise sequence identity, whereas lower accuracies are observed for the dataset having resistant proteins with <40% pair-wise sequence identity (Table 1 and Fig. 3). Though the specificities are observed almost unchanged, sensitivities are observed to be increased with increase in the percentage of pair-wise sequence identity in the positive dataset (Fig. 3). Besides, it is seen that the most of the performance metrics for DPC and CTD feature sets are higher as compared to the other feature sets (AAC, PAAC and ACF). In particular, overall accuracy (~90%), MCC (~89%) and AUC-ROC (~98%) are observed to be highest for DPC feature set. Since the number of sequences in the positive dataset having sequences with <90% pair-wise sequence identity is larger as compared to the dataset having sequences with <40% pair-wise sequence identity, the former one is used in subsequent analyses.

**Table 1:** Estimates of different performance metrics for SVM with RBF kernel in discriminating resistant from non-resistant proteins, under all the feature sets as well as different percentage of sequence identity in the positive dataset

		Performance metrics					
Id(%)	Feature	Sn	Sp	Ac	Pre	MCC	AUC-ROC
40	AAC	0.836 ± 0.018	0.952 ± 0.014	0.894 ± 0.012	0.946 ± 0.015	0.794 ± 0.024	0.924 ± 0.020
	DPC	0.849 ± 0.013	0.983 ± 0.011	0.916 ± 0.009	0.980 ± 0.012	0.839 ± 0.017	0.948 ± 0.011
	PAAC	0.836 ± 0.018	0.956 ± 0.014	0.896 ± 0.013	0.951 ± 0.015	0.798 ± 0.026	0.922 ± 0.018
	CTD	0.841 ± 0.015	0.981 ± 0.011	0.911 ± 0.010	0.978 ± 0.013	0.831 ± 0.020	0.932 ± 0.010
	ACF	0.836 ± 0.017	0.9530.016	0.895 ± 0.012	0.947 ± 0.017	0.795 ± 0.025	0.901 ± 0.017
60	AAC	0.870 ± 0.012	0.959 ± 0.008	0.914 ± 0.008	0.955 ± 0.009	0.832 ± 0.016	0.946 ± 0.008
	DPC	0.875 ± 0.008	0.986 ± 0.007	0.931 ± 0.006	0.984 ± 0.007	0.866 ± 0.011	0.972 ± 0.005
	PAAC	0.870 ± 0.014	0.960 ± 0.010	0.915 ± 0.010	0.956 ± 0.011	0.833 ± 0.020	0.947 ± 0.010
	CTD	0.860 ± 0.011	0.985 ± 0.007	0.923 ± 0.007	0.983 ± 0.008	0.852 ± 0.014	0.959 ± 0.006
	ACF	0.869 ± 0.011	0.964 ± 0.009	0.917 ± 0.007	0.960 ± 0.009	0.837 ± 0.015	0.932 ± 0.009
70	AAC	0.886 ± 0.011	0.961 ± 0.008	0.924 ± 0.008	0.958 ± 0.008	0.850 ± 0.015	0.953 ± 0.008
	DPC	0.883 ± 0.008	0.987 ± 0.005	0.935 ± 0.005	0.986 ± 0.005	0.875 ± 0.009	0.973 ± 0.004
	PAAC	0.891 ± 0.010	0.961 ± 0.008	0.926 ± 0.007	0.958 ± 0.008	0.854 ± 0.013	0.955 ± 0.007
	CTD	0.866 ± 0.010	0.987 ± 0.005	0.926 ± 0.006	0.985 ± 0.006	0.859 ± 0.012	0.961 ± 0.006
	ACF	0.888 ± 0.008	0.963 ± 0.009	0.925 ± 0.006	0.960 ± 0.009	0.853 ± 0.013	0.948 ± 0.007
90	AAC	0.886 ± 0.010	0.959 ± 0.006	0.923 ± 0.006	0.956 ± 0.006	0.847 ± 0.012	0.955 ± 0.006
	DPC	0.899 ± 0.009	0.989 ± 0.005	0.944 ± 0.006	0.988 ± 0.005	0.892 ± 0.011	0.978 ± 0.004
	PAAC	0.889 ± 0.011	0.959 ± 0.007	0.924 ± 0.007	0.956 ± 0.007	0.850 ± 0.014	0.956 ± 0.006
	CTD	0.887 ± 0.008	0.987 ± 0.005	0.937 ± 0.005	0.985 ± 0.006	0.878 ± 0.010	0.972 ± 0.005
	ACF	0.894 ± 0.010	0.967 ± 0.006	0.930 ± 0.006	0.964 ± 0.006	0.863 ± 0.013	0.949 ± 0.006

Id(%): maximum percentage of pair-wise sequence identity present in the positive dataset

*Sn* Sensitivity, *Sp* Specificity, *Ac* Accuracy, *Pre* Precision, *MCC* Matthew’s correlation coefficient, *AUC-ROC* area under ROC curves

### Analysis of mechanism-based classification

The values of performance metrics, with regard to classification of resistant proteins involved in target-based mechanism and detoxification-based mechanism, measured over LOOCV as well as 100 sample sets are given in Table 2. Performance metrics for all the feature sets are observed ≥90% and are found to be highest in case of DPC feature set. More specifically, overall accuracy for the DPC feature set is observed >97%, with >95% MCC and >97% AUC-ROC. Though the number of features for AAC and PAAC feature sets are almost same, classification accuracies for AAC feature set are seen to be higher than that of PAAC feature set. Since the sensitivity and specificity are >90%, it is inferred that hardly one sequence is misclassified in each category (as the number of sequences in each category is only 15).

**Table 2:** Estimates of performance metrics for classification of detoxification and target-based resistant proteins, under different feature sets

Feature	Sn	Sp	Ac	Pre	MCC	AUC-ROC
AAC	0.927 ± 0.020	0.966 ± 0.042	0.946 ± 0.024	0.966 ± 0.041	0.894 ± 0.049	0.960 ± 0.023
DPC	0.967 ± 0.067	0.985 ± 0.031	0.976 ± 0.035	0.986 ± 0.029	0.955 ± 0.065	0.972 ± 0.051
PAAC	0.929 ± 0.016	0.952 ± 0.048	0.941 ± 0.027	0.953 ± 0.046	0.883 ± 0.054	0.956 ± 0.028
CTD	0.895 ± 0.042	0.979 ± 0.035	0.937 ± 0.024	0.979 ± 0.035	0.879 ± 0.047	0.935 ± 0.036
ACF	0.912 ± 0.041	0.927 ± 0.051	0.919 ± 0.037	0.927 ± 0.049	0.840 ± 0.074	0.967 ± 0.021

*Sn* Sensitivity, *Sp* Specificity, *Ac* Accuracy, *Pre* Precision, *MCC* Matthew’s correlation coefficient, *AUC-ROC* area under ROC curves

### Discriminating target-based resistant proteins from non-resistant proteins

With regard to classification of target-based resistant proteins and non-resistant proteins, performance metrics over LOOCV and 100 sample sets (where each sample set consists of 15 target-based resistant proteins and 15 non-resistant proteins that were randomly drawn from the 3919 non-resistant proteins) are given in Table 3. The values of performance metrics are observed to be higher for DPC feature set. Specifically, accuracies in terms of all the performance metrics are observed ≥90% for DPC feature set, whereas the values of MCC and AUC-ROC for rest of the feature sets are observed to be <90%.

**Table 3:** Estimates of performance metrics for discriminating target-based resistant proteins from non-resistant proteins, under different features

Feature	Sn	Sp	Ac	Pre	MCC	AUC-ROC
AAC	0.912 ± 0.031	0.940 ± 0.055	0.926 ± 0.034	0.941 ± 0.052	0.854 ± 0.068	0.879 ± 0.045
DPC	0.924 ± 0.090	0.981 ± 0.041	0.952 ± 0.057	0.979 ± 0.043	0.909 ± 0.111	0.924 ± 0.083
PAAC	0.919 ± 0.029	0.947 ± 0.053	0.933 ± 0.034	0.948 ± 0.051	0.868 ± 0.067	0.880 ± 0.043
CTD	0.855 ± 0.037	0.945 ± 0.047	0.900 ± 0.034	0.941 ± 0.049	0.804 ± 0.069	0.844 ± 0.028
ACF	0.915 ± 0.037	0.927 ± 0.054	0.921 ± 0.037	0.928 ± 0.051	0.844 ± 0.074	0.846 ± 0.043

*Sn* Sensitivity, *Sp* Specificity, *Ac* Accuracy, *Pre* Precision, *MCC* Matthew’s correlation coefficient, *AUC-ROC* area under ROC curves

### Discriminating detoxification-based resistant proteins from non-resistant proteins

The classification was also made between 452 detoxification-based resistant proteins and 3919 non-resistant proteins, by using SVM with RBF kernel. Performances metrics were computed over 10 folds of cross validation as well as 100 sample sets (where each sample consists of 452 detoxification-based resistant proteins and 452 non-resistant proteins that were drawn randomly from the 3919 non-resistant proteins) are presented in Table 4. It is observed that the accuracies are higher for DPC feature set and lower for AAC feature set. In particular, the values of all the performances metrics for both CTD and DPC feature sets are ≥90% (Table 4). Barring sensitivity, the values of performance metrics in discriminating the detoxification-based resistant proteins from non-resistant proteins (Table 4) are higher as compared to that of discriminating target-based resistant proteins from non-resistant proteins (Table 3).

**Table 4:** Estimates of different performance metrics for discriminating detoxification-based resistant proteins from non-resistant proteins

Feature	Sn	Sp	Ac	Pre	MCC	AUC-ROC
AAC	0.898 ± 0.009	0.963 ± 0.006	0.931 ± 0.006	0.960 ± 0.007	0.863 ± 0.013	0.960 ± 0.007
DPC	0.911 ± 0.006	0.992 ± 0.004	0.951 ± 0.004	0.991 ± 0.004	0.905 ± 0.008	0.980 ± 0.004
PAAC	0.901 ± 0.008	0.965 ± 0.006	0.933 ± 0.006	0.962 ± 0.007	0.867 ± 0.012	0.960 ± 0.006
CTD	0.907 ± 0.007	0.990 ± 0.004	0.948 ± 0.005	0.989 ± 0.004	0.900 ± 0.009	0.974 ± 0.004
ACF	0.912 ± 0.007	0.969 ± 0.006	0.941 ± 0.005	0.968 ± 0.006	0.883 ± 0.010	0.959 ± 0.005

*Sn* Sensitivity, *Sp* Specificity, *Ac* Accuracy, *Pre* Precision, *MCC* Matthew’s correlation coefficient, *AUC-ROC* area under ROC curves

### Comparative analysis

For comparing the proposed approach with Blast algorithms, we prepared two different datasets. The *first* dataset contains 442 resistant proteins (with < 90% pair-wise sequence identity) and randomly drawn 442 non-resistant proteins (with <40% pair-wise sequence identity), and the *second* dataset contains 128 resistant proteins (with <40% pair-wise sequence identity) and randomly drawn 128 non-resistant proteins (with <40% pair-wise sequence identity). Furthermore, performance of the proposed approach was compared based on DPC feature set only as higher accuracies were obtained for this feature set as compared to the other feature sets. In both the datasets, no hits were found for most of the query sequences with e-values 0.1 and 1. However, hits were found for all the query sequences with e-value 10. Therefore, comparison was made based on e-value 10 only, and the accuracies averaged over 10-folds are given in Table 5. It is observed that the overall accuracies of the proposed approach are ~10% higher than that of Blastp, PSI-Blast and Delta-Blast, in both datasets (Table 5). Though, true positive rates (sensitivity) of the Blast algorithms are higher, false positive rates (specificity) are much lower at the same time. Among the Blast algorithms, Delta-Blast performed better than both Blastp and PSI-Blast, with both the datasets (Table 5). Barring sensitivity, the proposed approach performed better than Blast algorithms in terms of all the performance metrics. It is further seen that the specificities are higher for the *first* dataset as compared to the *second* dataset.

**Table 5:** Performance metrics for the proposed approach, Blast, PSI-Blast and Delta-Blast, in discriminating the resistant proteins from non-resistant proteins, where the positive dataset consists of <40% (first) and <90% (second) pair-wise sequence identity

Dataset	Method	Sn	Sp	Ac	Pre	MCC
First	Proposed	0.897	0.934	0.916	0.933	0.836
	Blast	0.961	0.611	0.786	0.713	0.617
	PSI-Blast	0.959	0.602	0.780	0.707	0.607
	Delta-Blast	0.961	0.652	0.806	0.735	0.647
Second	Proposed	0.875	0.891	0.883	0.901	0.784
	Blast	0.958	0.350	0.654	0.596	0.392
	PSI-Blast	0.958	0.358	0.658	0.601	0.400
	Delta-Blast	0.958	0.466	0.712	0.646	0.495

Here, AUC-ROC values were not computed, as in Blast algorithms accuracies are computed based on number of hits

*Sn* Sensitivity, *Sp* Specificity, *Ac* Accuracy, *Pre* Precision, *MCC* Matthew’s correlation coefficient

### Performance analysis based on independent test dataset

**Fig. 4:**
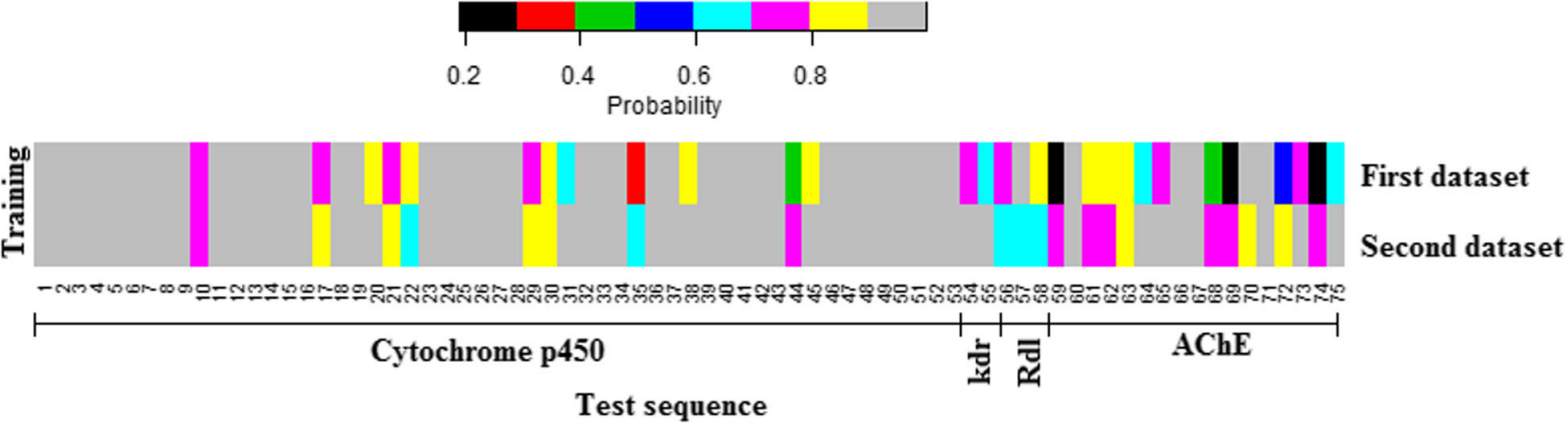
Heat map of the probabilities with which 75 test sequences are predicted in two different training datasets. All the 75 sequences are correctly predicted as resistant proteins in the second training dataset, whereas 69 are correctly predicted with the first training dataset. It is further seen that most of the test sequences are correctly predicted with high probabilities (>0.9)

Both the datasets mentioned in “comparative analysis” section were used to train the model for prediction of the level (as resistant or non-resistant) of each test sequence. Furthermore, none of the test sequences were present in the training set. It is observed that 69 out of 75 are correctly predicted while first dataset is used as training set (Table 6). On the other hand, all the 75 instances are correctly identified as insecticide resistant proteins with second dataset as training set (Table 6). Besides, it is seen that most of the sequences are correctly predicted with >0.9 probabilities irrespective of the training datasets (Fig. 4). More clearly, 2 test sequences of cytochrome P450 and 4 sequences of AChE are misclassified in the first training dataset (Fig. 4).

**Table 6:** Performance of the proposed approach based on an independent dataset of 75 insecticide resistant proteins

		Predicted	
Resistance family	Observed	1^st^ training model	2^nd^ training model
Cytochrome P450	53	51	53
Kdr	2	2	2
Rdl	3	3	3
AChE	17	13	17
Total	75	69	75

### Online prediction server: DIRProt

**Fig. 5:**
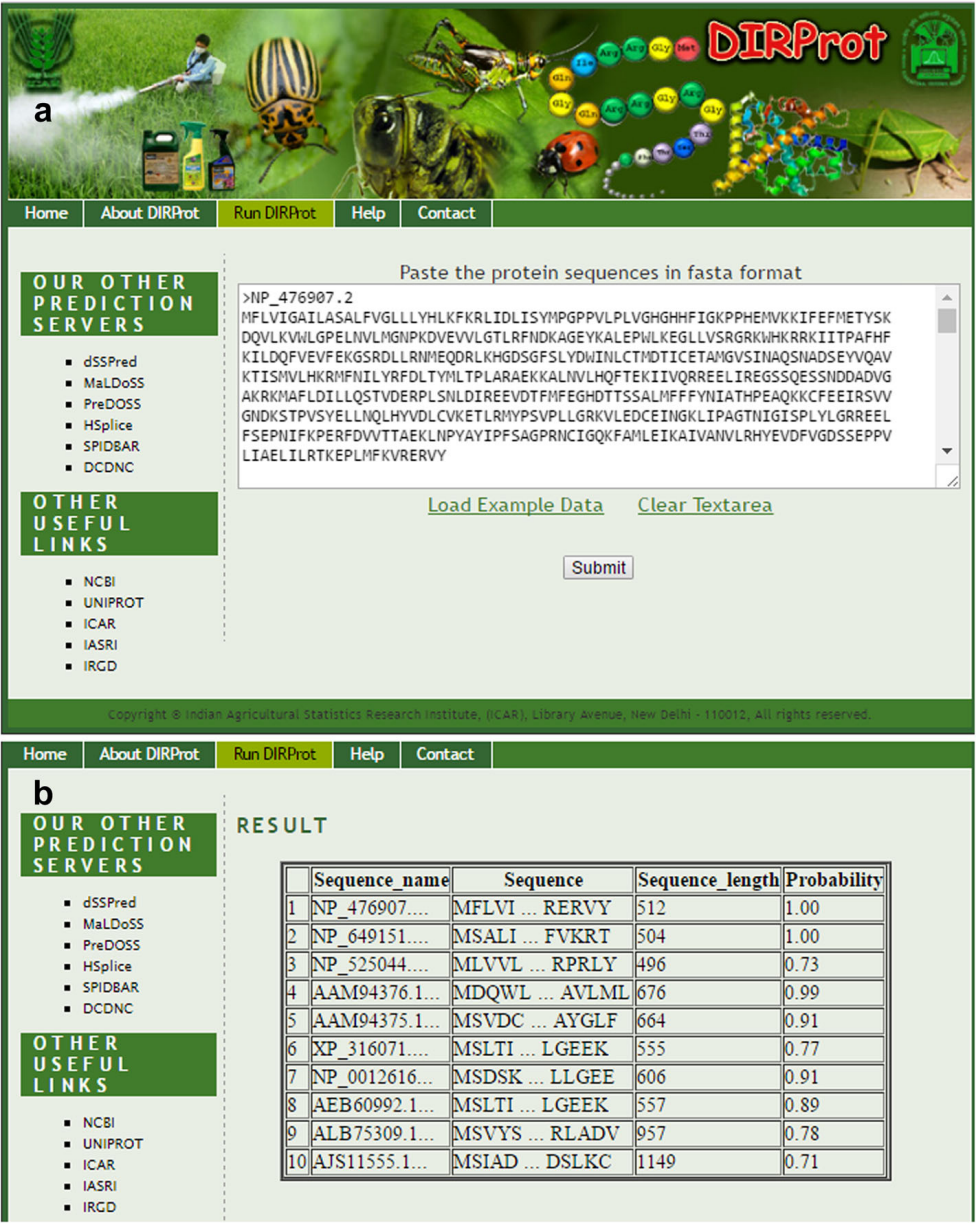
**a** Server page of DIRProt, **b** result page after execution with an example dataset. The result page is displayed in a tabular form, where the last column is the probabilities with which the each sequences are predicted as insecticide-resistant proteins

A web server DIRProt has been developed to discriminate the insecticide resistant proteins from non-resistant proteins. This server has been trained with the SVM (with RBF kernel) for prediction of insecticide resistant proteins based on DPC feature set. The web pages showing the execution and results for an example dataset are shown in Fig. 5a and b respectively. Help pages are also provided to guide the user regarding generation of features, prediction method and input–output. The sequences in FASTA format along with the annotations and probabilities with which they are predicted as resistance proteins are shown in the result page. For reproducible research, the trained datasets are also provided in the server. The prediction server is made freely accessible at http://cabgrid.res.in:8080/dirprot for academic users.

## Discussion

Extensive use of chemical insecticides has been selecting resistant population of insect species to different insecticides, worldwide [69, 70]. Around 590 insect species have been reported to resist different insecticides till the end of 2014 [71]. Insecticidal resistance has been associated with the genetic changes in insects. For instance, a mutation in an insect can alter the behavior, metabolism and physiology by which insect may gain advantage in resisting to different insecticides [70]. Most of the earlier studies are dealt with the mutational changes associated with the insecticide resistance. Though insecticide resistance is an important researchable issue, there is no computational tool available for prediction of insecticide resistant proteins. Therefore, we made an attempt to present the first computational approach for prediction of insecticide resistant proteins.

We considered four different categories of insecticide resistant proteins corresponding to four different classes of insecticide resistance genes viz., cytochrome P450, AChE, Rdl and Kdr. The leucine content was predominantly found in all the four categories of proteins, which has been reported to play an important role in insecticide resistance. For instance, Prince et al. [72] reported that leucine-rich repeat receptor-like kinase “brassinosteroid insensitive1-associated kinase1” contributes to the innate immunity to aphids in *Arabidopsis*. The valine to leucine (V419L) and the leucine to isoleucine mutations (L925I) were identified in three pesticide-resistant strains of bed bug (*Cimex lectularius*) [73]. Further, the composition of tryptophan which has been reported to present in the active site that interacts with trimethyl-ammonium cationic group of AchE was found lowest [73]. Hassani et al. [74] described that lysine and tryptophan (Lys12 and Trp39 and Trp54) are the most reactive residues that play important role in disrupting the function of neuronal sodium channels by Ts gamma, which is the most potent neurotoxin in the venom of the Brazilian scorpion *Tityus serrulatus.*

For classification of insecticide resistant and non-resistant proteins, initially the sequences were transformed into numeric feature vectors based on different feature generation techniques viz., AAC, DPC, PAAC, ACF and CTD. The encoded numeric vectors were then used as input in binary SVM classifier. Prediction accuracies were found to be higher for RBF kernel as compared to the other three kernels of SVM. Further, the classification accuracies were found higher for DPC feature set as compared to the other feature sets, which may be due to the fact that in DPC the local ordering of amino acids were taken into account [42, 43]. Furthermore, in cross validation analysis (Table 1), the sensitivity was found to be increased with increase in the percentage of pair-wise sequence identity in the positive dataset. This may be due to the fact that with increase in the pair-wise sequence identity in the positive dataset, it is less-likely that a positive sequence will be misclassified in the negative dataset. The accuracy in discriminating the target-based and detoxification-based resistance proteins from non-resistant proteins was also found to be higher. Besides, higher discrimination accuracy was also observed between target-based and detoxification-based resistance proteins. Thus, it can be inferred that the composition of di-peptides are not only different between resistant and non-resistant proteins but also among insecticide resistant proteins involved in different insecticide resistance mechanisms.

The performance of the proposed approach was compared with Blast, PSI-Blast and Delta-Blast algorithms. Though, prediction was made for three e-values i.e., 0.1, 1 and 10, no hits were found for most of the query sequences (particularly negative) for the first two e-values. Thus performance metrics were computed based on e-value 10 only, which is also the default e-value in Blast algorithms. Though the resistant proteins were predicted with higher accuracy, the specificities were found much lower. It was also found that the specificities are higher for the *first* dataset as compared to the *second* dataset. One of the possible reasons for this may be that when the pair-wise sequence identity is <40% in the positive class (first dataset), sequence similarity between the classes will be less. On the other hand, when the pair-wise sequence identity is <90% in the positive class, sequence similarity between the positive and negative classes will be more by which the likelihood of a sequence of the negative class to be predicted in the positive class will be more and vice versa. In terms of overall accuracy, the proposed approach outperformed all the three variations of Blast algorithm. Among the Blast algorithms, Delta-Blast performed better followed by PSI-Blast and Blast. The performance of the proposed approach was also assessed using an independent test dataset consisting of 75 resistant protein sequences (53 cytochrome P450, 2 Kdr, 3 Rdl and 17 AChE). Out these 75 sequences, all were correctly predicted when the pair-wise sequence identity was <90% in the positive dataset of training set, whereas 69 were correctly predicted in for the training dataset having positive sequences with <40% pair-wise sequence identity. Nevertheless, the proposed approach achieved higher accuracy for predicting the insecticide resistant proteins.

## Conclusions

This paper presents the first computational approach for predicting the insecticide resistant proteins. Based on this approach, a web server has also been developed that can be easily used by the scientists and researchers to computationally identify the insecticide resistant proteins. The proposed computational approach is believed to supplement the wet-lab experiments for identifying and targeting the insecticide resistant proteins to develop dynamic and efficient insecticides.

## Additional file

Additional file 1: It contains the list of insect species and corresponding insecticide resistant gene types that were used in this study. This file also contains the 75 insecticide resistant protein sequences that were used as independent dataset. (PDF 185 kb)

## Abbreviations

AAC: Amino acid composition
Ac: Accuracy
ACF: Auto correlation function
AChE: Acetylcholinesterases
AUC-ROC: Area under ROC curves
CTD: Composition-trainsition-distribution
DPC: Dipeptide composition
FN: False negative
FP: False positive
GABA: γ-amino butyric acid
GSTs: Glutathione S-transferases
Kdr: Knock down resistance
MCC: Matthew’s correlation coefficient
OSH: Optimal separating hyper-plane
PAAC: Pseudo amino acid composition
Pre: Precision
RBF: Radial basis function
Rdl: Resistance to dieldrin
ROC: Receiving operating characteristics
Sn: Sensitivity
Sp: Specificity
SVM: Support vector machine
TN: True negative
TP: True positive
