# Supplementary material for: DIRProt: a computational approach for discriminating insecticide resistant proteins from non-resistant proteins
Source: BMC Bioinformatics. 2017 Mar 24;18:190. doi: 10.1186/s12859-017-1587-y (PMC5364559; doi:10.1186/s12859-017-1587-y)
Supplement: Additional file 1: — It contains the list of insect species and corresponding insecticide resistant gene types that were used in this study. This file also contains the 75 insecticide resistant protein sequences that were used as independent dataset. (PDF 185 kb) [file 12859_2017_1587_MOESM1_ESM.pdf]

Summary of the insect species and the type of resistant genes

Insect species	Resistant genes
Helicoverpa armingera	Cytochrome P450
	Acetylcholinesterase
Aphis gossypii	Cytochrome P450
	KDR
Acyrthosiphon pisum	Cytochrome P450
	Acetylcholinesterase
Bemisia tabaci	Cytochrome P450
	Acetylcholinesterase
Plutella xylostella	Cytochrome P450
	RDL
Spodoptera exigua	Cytochrome P450
Spodoptera litura	Cytochrome P450
Nilaparavata lugena	Cytochrome P450
Myzus persicae	Cytochrome P450
Tribolium castaneum	Cytochrome P450
	RDL
Leucinodes orbonalis	Acetylcholinesterase

Independent test dataset

Cytochrome P450 (53)

>NP_610390.1 Cyp6a13 [Drosophila melanogaster]

MLTLLVLVFTVGLLLYVKLRWHYSYWSRRGVAGERPVYFRGNMSGLGRDLHWTDINLRIYRKFRGVERYC

GYFTFMTKSLFIMDLELIRDIMIRDFSSFADRGLFHNVRDDPLTGNLLFLDGPEWRWLRQNLTQVFTSGK

MKFMFPNMVEVGEKLTQACRLQVGEIEAKDLCARFTTDVIGSCAFGLECNSLQDPESQFRRMGRSVTQEP

LHSVLVQAFMFAQPELARKLRFRLFRPEVSEFFLDTVRQTLDYRRRENIHRNDLIQLLMELGEEGVKDAL

SFEQIAAQALVFFLAGFDTSSTTMSFCLYELALNPDVQERLRVEVLAVLKRNNQKLTYDSVQEMPYLDQV

VAETLRKYPILPHLLRRSTKEYQIPNSNLILEPGSKIIIPVHSIHHDPELYPDPEKFDPSRFEPEEIKAR

HPFAYLPFGEGPRNCIGERFGKLQVKVGLVYLLRDFKFSRSEKTQIPLKFSSRNFLISTQEGVHLRMEGL

ERP

>NP_652075.1 Cyp6a22, isoform A [Drosophila melanogaster]

MLDVVALLLIALAVGFWFVRTRYSYWTRRGIGSEPARFPVGNMEGFRKNKHFIDIVTPIYEKFKGNGAPF

AGFFMMLRPVVLVTDLELAKQILIQDFANFEDRGMYHNERDDPLTGHLFRIDGPKWRPLRQKMSPTFTSA

KMKYMFPTVCEVGEELTQVCGELADNAMCGILEIGDLMARYTSDVIGRCAFGVECNGLRNPEAEFAIMGR

RAFSERRHCKLVDGFIESFPEVARFLRMRQIHQDITDFYVGIVRETVKQREEQGIVRSDFMNLLIEMKQR

GELTIEEMAAQAFIFFAAGFDTSASTLGFALYELAKQPALQAKLREEIDQALRLHNGEFTYDSMQELRYM

ELVIAETLRKYPILPQLTRISRHLYAAKGDRHFYIEPGQMLLIPVYGIHHDPALYPEPHKFIPERFLADQ

LAQRPTAAWLPFGDGPRNCIGMRFGKMQTTIGLVSLLRNFHFSVCPRTDPKIEFLKSNILLCPANGIYLK

VQQLSQMSS

>NP_523628.1 cytochrome P450-6a2 [Drosophila melanogaster]

MFVLIYLLIAISSLLAYLYHRNFNYWNRRGVPHDAPHPLYGNMVGFRKNRVMHDFFYDYYNKYRKSGFPF

VGFYFLHKPAAFIVDTQLAKNILIKDFSNFADRGQFHNGRDDPLTQHLFNLDGKKWKDMRQRLTPTFTSG

KMKFMFPTVIKVSEEFVKVITEQVPAAQNGAVLEIKELMARFTTDVIGTCAFGIECNTLRTPVSDFRTMG

QKVFTDMRHGKLLTMFVFSFPKLASRLRMRMMPEDVHQFFMRLVNDTIALRERENFKRNDFMNLLIELKQ

KGRVTLDNGEVIEGMDIGELAAQVFVFYVAGFETSSSTMSYCLYELAQNQDIQDRLRNEIQTVLEEQEGQ

LTYESIKAMTYLNQVISETLRLYTLVPHLERKALNDYVVPGHEKLVIEKGTQVIIPACAYHRDEDLYPNP

ETFDPERFSPEKVAARESVEWLPFGDGPRNCIGMRFGQMQARIGLAQIISRFRVSVCDTTEIPLKYSPMS

IVLGTVGGIYLRVERI

>NP_523748.2 cytochrome P450-6a9 [Drosophila melanogaster]

MGVYSVLLAIVVVLVGYLLLKWRRALHYWQNLDIPCEEPHILMGSLTGVQTSRSFSAIWMDYYNKFRGTG

PFAGFYWFQRPGILVLDISLAKLILIKEFNKFTDRGFYHNTEDDPLSGQLFLLDGQKWKSMRSKLSYTFT

SGKMKYMFPTVVKVGHEFIEVFGQAMEKSPIVEVRDILARFTTDVIGTCAFGIECSSLKDPEAEFRVMGR

RAIFEQRHGPIGIAFINSFQNLARRLHMKITLEEAEHFFLRIVRETVAFREKNNIRRNDFMDQLIDLKNS

PLTKSESGESVNLTIEEMAAQAFVFFGAGFETSSTTMGFALYELAQHQDIQDRVRKECQEVIGKYNGEIT

YESMKDMVYLDQVISETLRLYTVLPVLNRECLEDYEVPGHPKYVIKKGMPVLIPCGAMHRDEKLYANPNT

FNPDNFSPERVKERDSVEWLPFGDGPRNCIGMRFGQMQARSGLALLINRFKFSVCEQTTIPIVYSKKTFL

ISSETGIFLKVERV

>NP_650224.3 Cyp313a4 [Drosophila melanogaster]

MLTWTLWCGLLFLLWIYFLWSRRRFYLLTLKIPGPLGYPILGMAHWLMRREDILNAFGCFLDKHGPTIFS

WLGPIPFMIVSDPQVVQDIFTSPHCVNKGIIYKAVDDGAGVGLFSLKDPRWSIHRKLLNPAFGHKVLLSF

LPIFNRETALLLDQLEPLQDDGEKDLIPLLQSFTLGIATQTTMGSDVKDEESFRSNSLLGRYQCILETMT

DMCFSPWLNSRFCRQLAGKESHYYQAKTEIRQFIRKIIERKLAEDEMGALPSIQSNDKNLFLNLVTDLMR

RGVFTLKNVEDESNIIVFGAFETTANAVYYTLMLLAMFPEYQERAFEEIKTIFPNTGDFDVSYADTQQMV

YLDLILNESMRVIPPVPVVSRQTSQDLKLSNGIVVPKGVQIAIDIYHMHRSKKIWGPDAETFNPDHFLPH

NIQDKHPYAYIPFTKGIRNCIGWRYALISAKVTLAKLLRNYRFKTSFPFENLYFVEDITMKLKSVPLLEL

QKRT

>NP_611000.2 Cyp6a23 [Drosophila melanogaster]

MSLLLTLIALLVSLLLFMARRRHGYWQRRGIPHDVPHPIYGNMKDWPKKRHIAMIFRDYYTKYKRSVYPF

AGFYFFFTRSAVITDLELVKRVLIKDFNHFENRGIFYNEIDDPLSATLFSIEGQKWRHLRHKLTPTFTSG

KMKNMFPIIVKVGEEMEKIFSAKTTTGEGQVLEIVDLVARYTADVIGNCAFGLNCNSLQNPNAEFVTIGK

RAIIERRYGGLLDFLIFGFPKLSRRLRLKLNVQDVEDFYTSIVRNTIDYRLRTNEKRHDFMDSLIEMYEK

EQAGNTEDGLSFNEILAQAFIFFVAGFETSSTTMGFALYELALDQDIQDQLRAEINNVLSKHNNEFTYEG

IKEMKYLEQVVMETLRKYPVLAHLTRMTQTDFSPEDPKYFIAKGTTVVIPALGIHYDPEIYPEPEKFKPE

RFTDEAIAARPSCTWLPFGEGPRNCIGLRFGLMQACVGLAYLIRGYKFSVSTETQIPMKFVVKSILLSAE

NGIHLKVEKLSK

>NP_610743.2 Cyp6g1, isoform A [Drosophila melanogaster]

MVLTEVLFVVVAALVALYTWFQRNHSYWQRKGIPYIPPTPIIGNTKVVFKMENSFGMHLSEIYNDPRLKD

EAVVGIYSMNKPGLIIRDIELIKSILIKDFNRFHNRYARCDPHGDPLGYNNLFFVRDAHWKGIRTKLTPV

FTSGKVKQMYTLMQEIGKDLELALQRRGEKNSGSFITEIKEICAQFSTDSIATIAFGIRANSLENPNAEF

RNYGRKMFTFTVARAKDFFVAFFLPKLVSLMRIQFFTADFSHFMRSTIGHVMEERERSGLLRNDLIDVLV

SLRKEAAAEPSKPHYAKNQDFLVAQAGVFFTAGFETSSSTMSFALYEMAKHPEMQKRLRDEINEALVEGG

GSLSYEKIQSLEYLAMVVDEVLRMYPVLPFLDREYESVEGQPDLSLKPFYDYTLENGTPVFIPIYALHHD

PKYWTNPSQFDPERFSPANRKNIVAMAYQPFGSGPHNCIGSRIGLLQSKLGLVSLLKNHSVRNCEATMKD
MKFDPKGFVLQADGGIHLEIVNDRLYDQSAPSLQ

>NP_476907.2 cytochrome P450-4d1, isoform A [Drosophila melanogaster]
MFLVIGAILASALFVGLLLYHLKFKRLIDLISYMPGPPVLPLVGHGHHFIGKPPHEMVKKIFEFMETYSK
DQVLKVWLGPELNVLMGNPKDVEVVLGTLRFNDKAGEYKALEPWLKEGLLVSRGRKWHKRRKIITPAFHF
KILDQFVEVFEKGSRDLLRNMEQDRLKHGDSGFSLYDWINLCTMDTICETAMGVSINAQSNADSEYVQAV
KTISMVLHKRMFNILYRFDLTYMLTPLARAEKKALNVLHQFTEKIIVQRREELIREGSSQESSNDDADVG
AKRKMAFLDILLQSTVDERPLSNLDIREEVDTFMFEGHDTTSSALMFFFYNIATHPEAQKKCFEEIRSVV
GNDKSTPVSYELLNQLHYVDLCVKETLRMYPSVPLLGRKVLEDCEINGKLIPAGTNIGISPLYLGRREEL
FSEPNIFKPERFDVVTTAEKLNPYAYIPFSAGPRNCIGQKFAMLEIKAIVANVLRHYEVDFVGDSSEPPV
LIAELILRTKEPLMFKVRERVY

>NP_649151.1 Cyp305a1 [Drosophila melanogaster]
MSALIFLCAILIGFVIYSLISSARRPKNFPPGPRFVPWLGNTLQFRKEASAVGGQHILFERWAKDFRSDL
VGLKLGREYVVVALGHEMVKEVQLQEVFEGRPDNFFLRLRTMGTRKGITCTDGQLWYEHRHFAMKQMRNV
GYGRSQMEHHIELEAEELLGQLERTEEQPIEPVTWLAQSVLNVLWCLIAGKRIARQEDGTLRRLLDLMNR
RSKLFDICGGLLAQFPWLRHVAPDRTGYNLIQQLNTELYGFFMDTIEEHRRQLAKDPSPAESDLIYAYLQ
EMKDRSAGGESSTFNETQLVMTILDFFIAGSQTTSNTINLALMVLAMRPDVQEKLFSQVTASVAAASTDA
FPHLSRREAFDYMDAFIMEVQRFFHITPITGPRRALWATKLGGYDIPKNATILISLRSVHLDKEHWKDPL
EFRPERFIDSAGKCFKDEYFMPFGMGRRRCLGDALARACIFSFLVRIVQHFSVVLPAGESPSMVLLPGIT
LTPKPYKVQFVKRT

>NP_525044.1 cytochrome P450-4ae1 [Drosophila melanogaster]
MLVVLLVALLVTRLVASLFRLALKELRHPLQGVVPSVSRVPLLGAAWQMRSFQPDNLHDKFAEYVKRFGR
SFMGTVLGHVVMVTAEPRHIDALLQGQHQLKKGTMYFALRGWLGDGLLLSRGKEWHTMRKIITPTFHFSI
LEQFVEVFDRQSSILVERLRTLSYGNEVVNIYPLVGLAALDIITETAMGVNVDAQGADSEVVHAVKDLTN
ILATRFMRPHLLFPHLFRLCWPSGFRKQQAGVICLHEFTNGIIEQRRRLLAREANQDKPTKPHALLDTLL
RATVDGQPLTDKQIRDEVNTFIFEGHDTTTSAVSFCLYLLSRHEAVQQKLFEELRMHYGQDLFRGVILSD
FATLPYLSCVVKESLRLYPPIPAVARCLEKDLVIDEGYIPVGTNVVVLLWQLLRDEAIFTDPLVFQPERH
LGEEAPRLSPYSYIPFSAGPRNCIGQKFALLEMKTMVTKVIRHYQLLPMGADVEPSIKIVLRSKSGVNVG
LRPRLY

>NP_611002.3 Cyp6a20 [Drosophila melanogaster]
MAVMIVLLIGVITFVAWYVHQHFNYWKRRGIPHDEPKIPYGNTSELMKTVHFADIFKRTYNKLRNKTDGP

FVGFYMYFKRMVVVTDIDFAKTVLIREFDKFHDRGVFHNERDDPLSANLVNIDGQKWKTLRQKLTPTFTS
GKMKTMFPTILTVGDELIRVFGETASADSDSMEITNVVARFTADVIGSCAFGLDCHSLSDPKAKFVQMGT
TAITERRHGKSMDLLLFGAPELAAKLRMKATVQEVEDFYMNIIRDTVDYRVKNNVKRHDFVDMLIEMKLK
FDNGDKENGLTFNEIAAQAFIFFLAGFETSSTTMGFALYELACHQDIQDKLRTEINTVLKQHNGKLDYDS
MREMTYLEKVIDETMRKRPVVGHLIRVATQHYQHTNPKYNIEKGTGVIVPTLAIHHDPEFYPEPEKFIPE
RFDEDQVQQRPACTFLPFGDGPRNCIGLRFGRMQVIVGMALLIHNFKFEFHPTKTVVPLEYRTDDFLLSS
KGGIHLKVTRV

>NP_611003.2 Cyp6a21 [Drosophila melanogaster]
MSVGTVLLTALLALVGYLLMKWRSTMRHWQDLGIPCEEPHILMGSMKGVRTARSFNEIWTSYYNKFRGSG
PFAGFYWFRRPAVFVLETSLAKQILIKEFNKFTDRGFFHNPEDDPLSGQLFLLDGQKWRTMRNKLSSTFT
SGKMKYMFPTVVKVANEFTDVFGQNVAKSPVVEVRELLARFTTDVIGTCAFGIECSSLKDPDAEFREMGR
RSLTEQRLGPVGIGFVNSFPNLARRLHMKMTAEPIERFFMRIVRETVAFREQNNIRRNDFMDQLIDLKNK
PLMVSQSGESVNLTIEEIAAQAFVFFAAGFETSSTTMGFALYELAQNQDIQNRVRKECQEVIEKCNGELN
YESMKDLVYLDQVVSETLRLYTVLPVLNRECLEDYEVPGHPKYVIKKGMPVLIPCGAMHRDEKLYANPNT
FNPDNFSPERVKERDSVEWLPFGDGPRNCIGMRFGQMQARIGLALLIKDFKFSVCEKTTIPMTYNKEMFL
IASNSGIYLKAERV

>NP_610744.1 Cyp6g2 [Drosophila melanogaster]
MELVLLILVASLIGIAFLALQQHYSYWRRMGVREIRPKWIVGNLMGLLNMRMSPAEFISQLYNHPDAENE
PFVGIHVFHKPALLLRDPEMVRNILVKDFAGFSNRYSSSDPKGDPLGSQNIFFLKNPAWKEVRLKLSPFF
TGNRLKQMFPLIEEVGASLDAHLRQQPLHNERMRCFDLEAKELCALYTTDVIATVAYGVSANSFTDPKCE
FRRHGRSVFEFNLLRAAEFTLVFFLPHLVPFVRFKVVPAEATRFLRKTINYVMSEREKSGQKRNDLIDIL
IEFRRSTQLAKASGIKDQFVFEGDILVAQAVLFFTAGFESSSSTMAFAMYELAKDTDVQQRLREEIKDAL
VESGGQVTLKMIESLEFMQMILLEVLRMYPPLPFLDRECTSGRDYSLAPFHKKFVVPKGMPVYIPCYALH
MDPQYFPQPRKFLPERFSPENRKLHTPYTYMPFGLGPHGCIGERFGYLQAKVGLVNLLRNHMITTSERTP
HRMQLDPKAIITQAKGGIHLRLVRDALGV

>NP_731751.1 Cyp304a1 [Drosophila melanogaster]
MITETLLTICAAVFLCLSYRYAVGRPSGFPPGPPKIPLFGSYLFMLIINFKYLHKAALTLSRWYKSDIIG
LHVGPFPVAVVHSADGVREILNNQVFDGRPQLFVAAMRDPGQDVRGIFFQDGPLWKEQRRFILRYLRDFG
FGRRFDQLELVIQEQLNDMLDLIRNGPKYPHEHEMVKSGGYRVLLPLLFNPFSANAHFYIVYNECLSREE
MGKLVKLCQMGIQFQRNADDYGKMLSIIPWIRHIWPEWSGYNKLNESNLFVRQFFADFVDKYLDSYEEGV
ERNFMDVYIAEMRRGPGYGFNRDQLIMGLVDFSFPAFTAIGVQLSLLVQYLMLYPAVLRRVQNEIDEVVG
CGRLPNLEDRKNLPFTEATIREGLRIETLVPSDVPHKALEDTELLGYRIPKDTIVVPSLYAFHSDARIWS

DPEQFRPERFLDADGKLCLKLDVSLPFGAGKRLCAGETFARNMLFLVTATMCQHFDFVLGPNDRLPDLSQ
NLNGLIISPPDFWLQLQDRH

>NP_650003.4 Cyp12e1 [Drosophila melanogaster]
MLSTQWNANKQISRQIYQLCRGLAQKATAVNLEEAKPYADIPGPSKLQLIRAFLPGGLYKNLPVHEMFLD
MNRQYGSIFRMPSVAGTDLVLTMNPQDYEVIFRNEGQYPYRRSFEVMDYFKRVHRREVFDGYDGLTSGNG
PAWGKMRTAVNPILLQPRNAKLYMTNLVQVSDEFLERIRIIRDPVTQEMPDDFAVDIRHLVIESICSVAL
NTHLGLLGEQRNNKDIQKLVLALQDVVELGFQLDIMPAFWKYLPMPNFKKLMRSLDTITDFCYFHIGNAL
KRIEEDAKAGTLNEIGLETSLLEKLARFDRQTAVIIAMDLLFAGADPTLVTLGGILFSLSKSPDKQARLL
EEIRGILPNKDSSLTIENMRNLPYLRACIKEGIRMYPIGPGTLRRMPHDVVLSGYRVVAGTDVGIAANYQ
MANMEQFVPKVREFIPERWLRDESNSHLVGETATPFMYLPFGFGPRSCAGKRIVDMMLEIAISRLVRNFK
IGFDYPIENAFKAQFFVQPNIPFKFKFIERNE

>NP_611067.2 Cyp4aa1 [Drosophila melanogaster]
MFVEKVLERTTNLELCSILILLVISLSIYTFYATLNTYLRSVLLSLRLTGPPSLPFLGNCMLVTDKDLMR
RCAGKAFDLYGSLVRIWVLLFPFFAVLEPEDLQVILSSKKHTNKVFFYRLMHNFLGDGLITSSGSKWSNH
RRLIQPAFHHNLLEKFIDTFVDASQSLYENLDAEAVGTEINIAKYVNNCVLDILNEAVLGVPIKKRGQDV
AMMEDSPFRQGKIMMPARFTQPWLLLDGIYHWTKMANDELNQKKRLNDFTRKMIQRRRQIQNNNNGNSER
KCLLDHMIEISESNRDFTEEDIVNEACTFMLAGQDSVGAAVAFTLFLLTQNPECQDRCVLELATIFEDSN
RAPTMTDLHEMRYMEMCIKEALRLYPSVPLIARKLGEEVRLAKHTLPAGSNVFICPYATHRLAHIYPDPE
KFQPERFSPENSENRHPYAFLPFSAGPRYCIGNRFAIMEIKTIVSRLLRSYQLLPVTGKTTIAATFRITL
RASGGLWVRLKERDHPLIAH

>NP_610252.3 Cyp6u1, isoform A [Drosophila melanogaster]
MDLMHRTLLTALGALSVVYALVKFSLGYWKRRGILHEKPKFLWGNIKGVVSGKRHAQDALQDIYTAYKGR
APFVGFYACLKPFILALDLKLVHQIIFTDAGHFTSRGLYSNPSGEPLSHNLLQLDGHKWRSLHAKSAEVF
TPANMQKLLVRLSQISSRIQRDLGEKSLQTINISELVGAYNTDVMASMAFGLVGQDNVEFAKWTRNYWAD
FRMWQAYLALEFPLIARLLQYKSYAEPATAYFQKVALSQLQLHRRRDRQPLQTFLQLYSNAEKPLTDIEI
AGQAFGFVLAGLGPLNATLAFCLYELARQPEVQDRTRLEINKALEEHGGQVTPECLRELRYTKQVLNETL
RLHTPHPFLLRRATKEFEVPGSVFVIAKGNNVLIPTAAIHMDPGIYENPQRFYPERFEEQARRSRPAAAF
LPFGDGLRGCIAARFAEQQLLVGLVALLRQHRYAPSAETSIPVEYDNRRLLLMPKSDIKLSVERVDKL

>NP_523961.1 cytochrome P450-4d8 [Drosophila melanogaster]
MQLMLRLNPKTFIKVGREYVLKFGHLQRVWIFNRLLIMSGDAELNEQLLSSQEHLVKHPVYKVLGQWLGN
GLLLSDGKVWHQRRKIITPTFHFSILEQFVEVFDQQSNICVQRLAQKANGNTFDVYRSICAAALDIIAET

AMGTKIYAQANESTPYAEAVNECTALLSWRFMSVYLQVELLFTLTHPHLKWRQTQLIRTMQEFTIKVIEK

RRQALEDQQSKLMDTADEDVGSKRRMALLDVLLMSTVDGRPLTNDEIREEVDTFMFEGHDTTTSALSFCL

HELSRHPEVQAKMLEEIVQVLGTDRSRPVSIRDLGELKYMECVIKESLRMYPPVPIVGRKLQTDFKYTHS

VHGDGVIPAGSEIIIGIFGVHRQPETFPNPDEFIPERHENGSRVAPFKMIPFSAGPRNCIGQKFAQLEMK

MMLAKIVREYELLPMGQRVECIVNIVLRSETGFQLGMRKRKHN

>NP_652018.1 cytochrome P450-6a17, isoform A [Drosophila melanogaster]

MLLLALIVVILSLLVFAARRRHGYWQRRGIPHDEVHPLFGNIKDWPNKRHIAEIFRDYYFKYKNSDYPFA

GFFFFFTRTAVVTDMELLKRVLIKDFNHFENRGVFYNEIDDPLSATLFSIEGQKWRHLRHKLTPTFTSGK

MKNMFPIVVKVGEEMDKVFRSKTAADRGQVLEVVDLVARYTADVIGNCAFGLNCNSLYDPKAEFVSIGKR

AITEHRYGNMLDIFLFGFPKLSRRLRLKLNIQEAEDFYTKIVRETIDYRLRTKEKRNDFMDSLIEMYKNE

QSGNSEDGLTFNELLAQAFIFFVAGFETSSTTMGFALYELARNQDVQDKLREEIGNVFGKHNKEFTYEGI

KEMKYLEQVVMETLRKYPVLAHLTRMTDTDFSPEDPKYFIAKGTIVVIPALGIHYDPDIYPEPEIFKPER

FTDEEIAARPSCTWLPFGEGPRNCIGLRFGMMQTCVGLAYLIRGYKFSVSPETQIPMKIVVKNILISAEN

GIHLKVEKLAK

>NP_611004.1 Cyp317a1, isoform A [Drosophila melanogaster]

MWIIFLIIGLLVLGLLVLLIIAARYQRDYWRYLDIPHERPKKLWPIIRQIMTQTLSTEAMKAEHYSAIYK

KFKGSGPFCGFYALLQPRALILDRELIRQIMIKDFWNFNDRGLYCNQKSDPLSGDLYALRGESWKEMRQK

LDPSLEGDRMSLLYDCLYEEAEQLLLTVNSTLMSQPHSTVHIQKIMRRYVLSSLAKCVFGLNAEQRKTYP

LEDFEQMTELALNSHKHGYLMNLMMIRFPNFCRMLRMRRTPKQAEEYFIKLLTSIVEQRETSGKPQKDYL

QLLIDVKALEFITHQYEADKELGAHLQNELAAHAVVFLKAGYEQTANTLSYVLYELALHPELQVRVREEV

KKAIERHDGHITHEGIKSLSFMGQVINETLRMHPITPYILRRTLNDYAVPDHPKYILVKELFLIIPTHAI

HHDPDIYPDPEEFKPDRWSGPRDSLQEQGTWFGFGVGARSCIGIQFAQLQLRLALALLLSEYEFSLNTRK

PLINLEDGIALTLMPLGVIEPGNEERAV

>NP_608912.1 Cyp28d1 [Drosophila melanogaster]

MCPISTALFVIAAILALIYVFLTWNFSYWKKRGIPTAKSWPFVGSFPSVFTQKRNVVYDIDEIYEQYKNT

DSIVGVFQTRIPQLMVTTPEYAHKIYVSDFRSFHDNEMAKFTDSKTDPILANNPFVLTGEAWKERRAEVT

PGLSANRVKAAYPVSLRVCKKFVEYIRRQSLMAPAQGLNAKDLCLCYTTEVISDCVLGISAQSFTDNPTP

MVGMTKRVFEQSFGFIFYTVVANLWPPITKFYSVSLFAKDVAAFFYDLMQKCIQVRRESPAAQQRDDFLN

YMLQLQEKKGLNAAELTSHTMTFLTDGFETTAQVLTHTLLFLARNPKEQMKLREEIGTAELTFEQISELP

FTEACIHETLRIFSPVLAARKVVTEPCELTNKNGVSVKLRPGDVVIIPVNALHHDPQYYEEPQSFKPERF

LNINGGAKKYRDQGLFFGFGDGPRICPGMRFSLTQIKAALVEIVRNFDIKVNPKTRKDNEIDDTYFMPAL

KGGVWLDFVERN

>NP_608403.1 Cyp6v1, isoform A [Drosophila melanogaster]

MVYSTNILLAIVTILTGVFIWSRRTYVYWQRRRVKFVQPTHLLGNLSRVLRLEESFALQLRRFYFDERFR

NEPVVGIYLFHQPALLIRDLQLVRTVLVEDFVSFSNRFAKCDGRSDKMGALSLFLAKQPEWREIRTRLAP

AFAGAKLKQMFSLMEEIGCDLEWYLKRLTRDLRRGDAERGAIVSIKDVCDLYNTDMIASIAFGLRSYSLR

NTQSEIGSHCQDLFRPNVRRIIDLFVIFYLPKLVPLLRPKLFTEPHAEFLRRVIQLVIEERERGGDLRND

LIEMLLTLKKEADLQQDKSHFTHHRDFLAAQAASFEVAGIETCSASMSFALYELAKQPLMQSRLRREIRE

AFASNPNGRLTYEAVARMEFLDMVVEETLRKYPIVPLLERECTPINKKRFYSLRPHAECYTRRGMPVFIS

NLAIHHDPKYWPDPDRFDPERFSAANKALQAPMSYMPFGAGPRNCIGMQIGLLQIKLGLVYFLHQHRVEI

CDRTVERIQFDAKFALLASEQRIYLKVDCL

>NP_525043.1 cytochrome P450-4d2, isoform A [Drosophila melanogaster]

MLGVVGVLLLVAFATLLLWDFLWRRRGNGILPGPRPLPFLGNLLMYRGLDPEQIMDFVKKNQRKYGRLYR

VWILHQLAVFSTDPRDIEFVLSSQQHITKNNLYKLLNCWLGDGLLMSTGRKWHGRRKIITPTFHFKILEQ

FVEIFDQQSAVMVEQLQSRADGMTPINIFPVICLTALDIIAETAMGTKINAQKNPNLPYVQAVNDVTNIL

IKRFIHAWQRVDWIFRLTQPTEAKRQDKAIKVMHDFTENIIRERRETLVNNSKETTPEEEVNFLGQKRRM

ALLDVLLQSTIDGAPLSDEDIREEVDTFMFEGHDTTTSAISFCLYEISRHPEVQQRLQQEIRDVLGEDRK

SPVTLRDLGELKFMENVIKESLRLHPPVPMIGRWFAEDVEIRGKHIPAGTNFTMGIFVLLRDPEYFESPD

EFRPERFDADVPQIHPYAYIPFSAGPRNCIGQKFAMLEMKSTVSKLLRHFELLPLGPEPRHSMNIVLRSA

NGVHLGLKPRA

>NP_525031.1 cytochrome P450-4g1 [Drosophila melanogaster]

MAVEVVQETLQQAASSSSTTVLGFSPMLTTLVGTLVAMALYEYWRRNSREYRMVANIPSPPELPILGQAH

VAAGLSNAEILAVGLGYLNKYGETMKAWLGNVLLVFLTNPSDIELILSGHQHLTKAEEYRYFKPWFGDGL

LISNGHHWRHHRKMIAPTFHQSILKSFVPTFVDHSKAVVARMGLEAGKSFDVHDYMSQTTVDILLSTAMG

VKKLPEGNKSFEYAQAVVDMCDIIHKRQVKLLYRLDSIYKFTKLREKGDRMMNIILGMTSKVVKDRKENF

QEESRAIVEEISTPVASTPASKKEGLRDDLDDIDENDVGAKRRLALLDAMVEMAKNPDIEWNEKDIMDEV

NTIMFEGHDTTSAGSSFALCMMGIHKDIQAKVFAEQKAIFGDNMLRDCTFADTMEMKYLERVILETLRLY

PPVPLIARRLDYDLKLASGPYTVPKGTTVIVLQYCVHRRPDIYPNPTKFDPDNFLPERMANRHYYSFIPF

SAGPRSCVGRKYAMLKLKVLLSTIVRNYIVHSTDTEADFKLQADIILKLENGFNVSLEKRQYATVA

>NP_524828.1 cytochrome P450-4p1 [Drosophila melanogaster]

MIILWLILALSALLYWLHRANKDYHILSFFTKRIRLKDGTPVEIIAPIAKGKTIFGNTLDLYGRDHAGVF

NYSRERAKEMGTSYIEYVFGKAIYNIIDADSAENVLNHPNLITKGLVYNFLHPFLRTGLLTSTGKKWHAR

RKMLTPTFHFNILNQFQEIFKTESQKFLLQFEGQDEVTITLHDVIPRFTLNSICETAMGVKLDEMAEKGD

RYRENFSQIEECFIRRLSNPLLWGDKLFEMFAAKDFASALDVVHRFSSEIIAKRRDLLKDELDKSSSTAD
DDGFVSKKRFAMLDTLIYAEKDGLIDHIGICEEVDTLMFEGYDTTSIGLIFGLMNMSLNPDKQELCYQEI
QEHIDDDLSNLDVGQLNKLKYLEYFMKETTRLFPSVPIMGREAVQETELANGLILPKGAQITIHVFDIHR
NAKYWDSPEEFRPERFLPENVQDRHTYAYVPFSAGQRNCIGKKYAMQEMKTLMVVLLKQFKVLKAIDPQK
IVFHTGITLRTQDKIRVKLVRRT

>NP_524598.1 cytochrome P450-4c3 [Drosophila melanogaster]
MSSKVITSLMAESILLSKVGQVISGYSPITVFLLGSILIFLVVYNKRRSRLVKYIEKIPGPAAMPFLGNA
IEMNVDHDELFNRVIGMQKLWGTRIGINRVWQGTAPRVLLFEPETVEPILNSQKFVNKSHDYDYLHPWLG
EGLLTSTDRKWHSRRKILTPAFHFKILDDFIDVFNEQSAVLARKLAVEVGSEAFNLFPYVTLCTLDIVCE
TAMGRRIYAQSNSESEYVKAVYGIGSIVQSRQAKIWLQSDFIFSLTAEYKLHQSYINTLHGFSNMVIRER
KAELAILQENNNNNNNNAPDAYDDVGKKKRLAFLDLLIDASKEGTVLSNEDIREEVDTFMFEGHDTTSAA
ISWTLFLLGCHPEYQERVVEELDSIFGDDKETPATMKNLMDMRYLECCIKDSLRLFPSVPMMARMVGEDV
NIGGKIVPAGTQAIIMTYALHRNPRVFPKPEQFNPDNFLPENCAGRHPFAYIPFSAGPRNCIGQKFAILE
EKAVISTVLRKYKIEAVDRREDLTLLGELILRPKDGLRVKITPRD

>NP_523646.1 cytochrome P450-9b2 [Drosophila melanogaster]
MALIEICLALVVIGYLIYKWSTATFKTFEERKLYFEKPYPFVGNMAAAALQKSSFQRQLTEFYERTRQHK
LVGFFNMRTPMITLNDPELIKKVCVKDFDHFPNHQPFITSNDRLFNDMLSVMRDQRWKHMRNTLTPVFTA
AKMRNMFTLMNESFAECLQHLDSSSKTLPGRKGFEVDMKVMCNKLSNDIIATTAFGLKVNSYDNPKNEFY
EIGQSLVFSRGLQFFKFMLSTLVPKLFSLLKLTIFDSAKVDYFARLVVEAMQYREKHNITRPDMIQLLME
AKNESEDKWTDDEIVAQCFIFFFAAFENNSNLICTTTYELLYNPDVQERLYEEIVETKKALNGAPLTYDA
VQKMTYMDMVISESLRKWTLAAATDRLCSKDYTLTDDDGTKLFDFKVGDRINIPISGLHLDDRYFPEPRK
FDPDRFSEERKGDMVPYTYLPFGVGPRNCIGNRYALMQVKGMLFNLLLHYKIEASPRTIKDLWGSASGFN
FTPRSGFWMHLVPRK

>NP_611370.2 Cyp12b2 [Drosophila melanogaster]
MWKYSNKIIYRNVSGNQLWFNRNSSVGGTLSQQTQLELADSRIDEKWQQARSFGEIPGPSLLRMLSFFMP
GGALRNTNLIQMNRLMREMYGDIYCIPGMMGKPNAVFTYNPDDFEMTYRNEGVWPIRIGLESLNYYRKIH
RPDVFKGVGGLASDQGQEWADIRNKVNPVLMKVQNVRQNLPQLDQISKEFIDKLETQRNPETHTLTTDFH
NQLKMWAFESISFVALNTRMGLLSDNPDPNADRLAKHMRDFFNYSFQFDVQPSIWTFYKTAGFKKFLKTY
DNITDITSNYIETAMRGFGKNDDGKTKCVLEQLLEHNKKVAVTMVMDMLMAGIDTTSSACLTILYHLARN
PSKQEKLRRELLRILPTTKDSLTDQNTKNMPYLRACIKEGLRITSITPGNFRITPKDLVLSGYQVPRGTG
VLMGVLELSNDDKYFAQSSEFIPERWLKSDLAPDIQACPAARTRNPFVYLPFGFGPRTCIGKRIAELEIE
TLLVRLLRSYKVSWLPETPIEYESTIILSPCGDIRFKLEPVGDLM

>NP_608689.2 Cyp309a2 [Drosophila melanogaster]

MMPERFSFDFRPIDQYWTRAKGACSNTKRGNMYILASLALILLHLLVLPIYLYLTWHHKYWRKRGLVTAR
PLTLLGTYPGLLTRKSNLVFDVQKIYDKYKGKHRAVGVFVTRQPQILVLDPELAHEVLVSNFRCYKDSLQ
SSYLRHAKWDKYARLNPFWASGQSWRRLRTDAQAGISGSRLRQAYNIWEQGGQMLTEYMTQQVAEKNNIL
ETRDLCFRYTAHVMADFIWGIDAGTLTRPMEQPNKVQEMASKWTSYAFYMLTLFMATIVAPCSRLLLRFR
FYPKETDEFFSNLTKESIELRLKAGDSTRTDYLSHLLQLRDQKQATHDDLVGHALTVMLDGYDTSGTALL
HALYYLAENPAVQQKLRVEILSCMASEKSLDFEKLSSLQYLEQVIYESLRLSSLIPQYTKVCTLPTVIRL
SESKSLDVEVGMTIMIPNYQFHHDKQYFPEPEAFKPERFDNGAYQELMRKGIFLPFSDGPRICMGVPLAM
LTLKSALVHILSNFQVVRGRDRLIPKGDSGFGVVLQGDVNLEYRRFFR

>NP_572780.3 Cyp311a1 [Drosophila melanogaster]

MALWPLLLITLTIWILVRKWTLLRLGSSLPGPWAFPLLGNAQMVGKLRPEYIFLVFTELRDRFGATYRLR
LGPQLWVFLHSAEETRQALHDPTLRKADTFMQLEPLIGNGLLISHGAHWTRQRRLLTPAFQPQLLRSFAP
AIGGHVERLVGRLGATRGAFLEVTEPLFACLLDAIVDTSMGAQLDTQSVDHSPIIQAFHLSSKLLFKRMI
NPLLSSDWIFQRTQLWRDLDEQLQVIHSQMESVIEKRAKELLDMGEPAGRAHNLLDTLLLAKFEGQSLSR
REIRDEINTFVFAGVDTTTAAMSFVLYALAKFPETQTRLRKELQDVALDETTDLDALNGLPYLEALIKEV
LRLYTIVPTTGRQTTQSTEIGGRTYCAGVTLWINMYGLAHDKEYYPDPYAFKPERWLPEDGAVAPPAFSY
IPFSGGPHVCIGRRYSLLLMKLLTARLVREFQMELSPEQAPLRLEAQMVLKAQQGINVSFLKQ

>NP_647975.2 spook, isoform A [Drosophila melanogaster]

MLAALIYTILAILLSVLATSYICIIYGVKRRVLQPVKTKNSTEINHNAYQKYTQAPGPRPWPIIGNLHLL
DRYRDSPFAGFTALAQQYGDIYSLTFGHTRCLVVNNLELIREVLNQNGKVMSGRPDFIRYHKLFGGERSN
SLALCDWSQLQQKRRNLARRHCSPREFSCFYMKMSQIGCEEMEHWNRELGNQLVPGEPINIKPLILKACA
NMFSQYMCSLRFDYDDVDFQQIVQYFDEIFWEINQGHPLDFLPWLYPFYQRHLNKIINWSSTIRGFIMER
IIRHRELSVDLDEPDRDFTDALLKSLLEDKDVSRNTIIFMLEDFIGGHSAVGNLVMLVLAYIAKNVDIGR
RIQEEIDAIIEEENRSINLLDMNAMPYTMATIFEVLRYSSSPIVPHVATEDTVISGYGVTKGTIVFINNY
VLNTSEKFWVNPKEFNPLRFLEPSKEQSPKNSKGSDSGIESDNEKLQLKRNIPHFLPFSIGKRTCIGQNL
VRGFGFLVVVNVMQRYNISSHNPSTIKISPESLALPADCFPLVLTPREKIGPL

>NP_523749.2 cytochrome P450-6a8 [Drosophila melanogaster]

MALTYILFQVAVALLAILTYYIHRKLTYFKRRGIPFVAPHLIRGNMEELQKTKNIHEIFQDHYNKFRESK
APFVGFFFFQSPAAFVIDLELAKQILIKDFSNFSNKGIFYNEKDDPISAHLFNLDGAQWRLLRNKLSSTF
TSGKMKLMYPTVVSVANEFMTVMHEKVPKNSVLEIRDLVARFTVDVIGTCAFGIQCNSLRDEKAEFLYFG
KRSLVDKRHGTLLNGFMRSYPKLARKLGMVRTAPHIQEFYSRIVTETVAVREKEHIKRNDFMDMLIELKN

QKEMTLENGDVVRGLTMEEVLAQAFVFFIAGFETSSSTMGFALYELAKNPDIQDKVRAEVEEVIEQHDQN
FTYECTKDLKYLNQVLDETLRLYTIVPNLDRMAAKRYVVPGHPNFVIEAGQSVIIPSSAIHHDPSIYPEP
FEFRPERFSPEESAGRPSVAWLPFGDGPRNCIGLRFGQMQARIGLALLIRNFKFSTCSKTPNPLVYDPKS
FVLGVKDGIYLKVETV

>NP_724937.1 Cyp49a1, isoform C [Drosophila melanogaster]
MELRPSMPSLRHYKGDLRRDFFGDVAGLIGVHGPKWEAFRQEVQHILLQPQTAKKYIPPLNDIASEFMGR
IELMRDEKDELPANFLHELYKWALESVGRVSLDTRLGCLSPEGSEEAQQIIEAINTFFWAVPELELRMPL
WRIYPTKAYRSFVKALDQFTAICMKNIGKTMDKADADEARGLSKSEADISIVERIVRKTGNRKLAAILAL
DLFLVGVDTTSVAASSTIYQLAKNPDKQKKLFDELQKVFPHREADINQNVLEQMPYLRACVKETLRMRPV
VIANGRSLQSDAVINGYHVPKGTHVIFPHLVVSNDPAYFPEPKRFLPERWLKQSTDAAGCPHANQKIHPF
VSLPFGFGRRMCVGRRFAEIELHTLLAKIFRKYKVSYNSGEFVYRVNSTYIPQSPLNFKLTLRDE

>NP_573003.2 Cyp4s3 [Drosophila melanogaster]
MSTLALVAFVLWAAFLRYLPKILNFLRLQRFAKTLPGPTIGELIANVKKGEILNWLKELREKHGPVFRIW
FGKDLMVMFTDPEDIKQLLGNNQLLTKSRNYELLEPWLGKGLLTNGGESWHRRRKLLTPGFHFRILSEFK
EPMEENCRILVRRLRTKANGESFDIYPYITLFALDAICETAMGIKKHAQLQSDSEYVQAVQSICRVMHKQ
SFSFWQRLNVFFKHTKPGKEREAALKVLHDETNRVIRLRREQLIQERNEWKPEAEQDDVGAKRRLAFLDM
LLLTQMEGGAELSDTDIREEVDTFMFEGHDTTSSAIAFALSLLSKNPDVQQRAFEEASELEGREKESMPY
LEAVIKETLRIYPSVPFFSRKVLEDLEVGKLTVPKGASISCLIYMLHRDPKNFPDPERFDPDRFLVNEKQ
MHPFAFAAFSAGPRNCIGQKFAMLELKTSLAMLLRSYRFLPDKDHQPKPLAELVTKSGNGIRLRILPRDE
NGTTA

>NP_727590.1 Cyp318a1 [Drosophila melanogaster]
MHLNIALWACGALLAVLLAWQQRKCWRLIWQLNGWRGVIQQPVLWLLLCINLHPNSILEKVSQYRVHFQR
PLAVLVGTRVLLYIDDPAGMECVLNAPECLDKTFLQDGFFVRRGLLHARGQKWKLRRKQLNPAFSHNIVA
SFFDVFNSVGNQMVEQFQTQTNLHGQAVKFTAAEDLLSRAVLEVSCLTIMGTPTNFTQLDDAHIAHSYKR
LLEISAVRVVKPWLQIRLLHRLLAPELYEESKKCAKLLEDFVGGIVRTKHRNWRLRDAVGGEKSGEDASN
GWQRRIFIEQIFQLAANGEMTLEEIMDEAQSMVLVSFETVSNSIMLALLCLATNKGDCQRRLLAEIRALV
PDVGQVGLEQLQQLRYLDAFVSESLRLLATVPMNLRHVSRDFRLAGRQHETIVPQNSIVVLDTFNMQRDE
RWWGANARQFDPQRFLDQEEEQLSKGHNDSGSGEKRRQRDRRHSYSFLPFSNGLRSCIGRRYGLFIMKVF
LVKLITNFDFQSDFELEKLQFVENISLKFKNADDILLTIQPKKEST

>NP_477117.2 cytochrome P450-4e2, isoform A [Drosophila melanogaster]
MWFVLYIFLALPLLLVAYLELSTFRRRRVLNKFNGPRGLPLMGNAHQMGKNPSEILDTVFSWWHQYGKDN

FVFWIGTYSNVLVTSSKYLEFILSSQTLITKSDIYQLTHPWLGLGLLTSTGSKWHKHRKMITPAFHFNIL
QDFHEVMNENSTKFIKHLKTVAAGDNIFDFQEQAHYLTLDVICDTAMGVSINAMENRSSSIVQAFKDMCY
NINMRAFHPLKRNELLYRLAPDYPAYSRTLKTLQDFTNEIIAKRIEAHKSGAVSTNAGDEFTRKKMAFLD
TLLSSTIDGRPLNSKELYEEVSTFMFEGHDTTTSGVSFAVYLLSRHQDEQRKLFKEQREVMGNSELGRDA
TFQEISQMKYLDLFIKEAQRVYPSVPFIGRFTEKDYVIDGDLVPKGTTLNLGLVMLGYNEKVFKDPHKFR
PERFELEKPGPFEYVPFSAGPRNCIGQKFALLEIKTVVSKIIRNFEVLPALDELVSKDGYISTTIGLPDA
ERKKRDPYRHKYDPILSAVLTLKSENGLYIRLKERH

>NP_650368.1 Cyp313a1 [Drosophila melanogaster]
MLTINLLLAVGALFWIYFLWSRRRLYFLMLKIPGPIGLPILGSSLENIITYKRKLSFRTKYLNKYGSTIL
TWMGPVPFIVTRDPKVVEDIFSSPDCHNKSQHIVNAITSCMGNGLLGKQDPHWLDRRKHFNPSFKQDLLL
SFFHIFDAETKVLMNLLDTYVDKGEIDVVPEMLRWSFKIAAQTTMGSEVKHDEHFKNGSLVESFESLISH
STLNILMPLVQNRMISKICGYDKLRADNFSRIQKMLDNVVNKKVNPLPKTDSDPESNIVINRAMELYRKG
DITYMDVKSECCIMIAAGYDTSALTVYHALFLLANHPEHQEAVFEELNGVFPDAGHFGITYPDMQKLDYL
ERVIKETLRLIPAIPITARETKNDVRLSNGVLIPKGVVIGIDMFHTHRNPEVWGPDADNFNPDNFLAENM
EQKHPYAYIPFARGKRNCIGSKYAMMSSKFALCRILRNYKISTSTLYKDLVYVDNMTMKLAEYPRLKLQR
RG

>NP_650327.1 Cyp6d5 [Drosophila melanogaster]
MIGIYLLIAAVTLLYVYLKWTFSYWDRKGFPSTGVSIPFGALESVTKGKRSFGMAIYDMYKSTKEPVIGL
YLTLRPALLVRDAQLAHDVLVKDFASFHDRGVYVDEKNDPMSASLFQMEGASWRALRNKLTPSFTSGKLK
AMFETSDSVGDKLVDSIRKQLPANGAKELELKKLMATYAIDIIATTIFGLDVDSFADPNNEFQIISKKVN
RNNIEDIIRGTSSFLYPGLEKFFVKIGWKQEATERMRELSNRTVDLREQNNIVRKDLLQLLLQLRNQGKI
NTDDNIWSAESTKNGVKSMSKDLIAGQLFLFYVAGYETTASTTSFTLYELTQNPEVMEKAKEDVRSAIEK
HGGKLTYDAISDMKYLEACILETARKYPALPLLNRICTKDYPVPDSKLVIQKGTPIIISLIGMHRDEEYF
PDPLAYKPERYLENGKDYTQAAYLPFGEGPRMCIGARMGKVNVKIAIAKVLSNFDLEIRKEKCEIEFGVY
GIPLMPKSGVPVRLSLKK

>NP_651563.1 Cyp6a18, isoform A [Drosophila melanogaster]
MQLTYFLFQVAVALLAIVTYILHRKLTYFKRRGIPYDKPHPLRGNMEGYKKTRTVHEIHQEYYNKYRNSK
APFVGFYLFQKPAAFVIDLELAKQILIKNFSNFTDKGIYYNEKDDPMSAHLFNLDGPQWRLLRSKLSSTF
TSGKMKFMYPTVVSVAEEFMAVMHEKVSENSILDVRDLVARFTVDVIGTCAFGIKCNSLRDEKAEFLHFG
RRALLDSRHGNLVSGLMRSYPNLARRLGLCRNTAQIQEFYQRIVKETVTLREKENIKRNDFMDMLIGLKN
QKNMTLENGEVVKGLTMDEIVAQAFVFFIAGFDTSSSTMGFALYELAKNPSIQDKVRAELGQVLEQHDQK
FTYECIKDLKYLDQVINETLRHYTIVPNVDRVAAKRFVVPGNPKFVIEAGQSVIIPSSAIHHDPSIYPEP

NEFRPERFSPEESAKRPSVAWLPFGEGPRNCIGLRFGQMQARIGLAMLIKNFTFSPCSATPDPLTFDPHS
AILLGIKGGIQLKVEAI

>NP_650189.1 Cyp9f2 [Drosophila melanogaster]
MLWEFFALFAIAAALFYRWASANNDFFKDRGIAYEKPVLYFGNMAGMFLRKRAMFDIVCDLYTKGGSKKF
FGIFEQRQPLLMVRDPDLIKQITIKDFDHFINHRNVFATSSDDDPHDMSNLFGSSLFSMRDARWKDMRST
LSPAFTGSKMRQMFQLMNQVAKEAVDCLKQDDSRVQENELDMKDYCTRFTNDVIASTAFGLQVNSFKDRE
NTFYQMGKKLTTFTFLQSMKFMLFFALKGLNKILKVELFDRKSTQYFVRLVLDAMKYRQEHNIVRPDMIN
MLMEARGIIQTEKTKASAVREWSDRDIVAQCFVFFFAGFETSAVLMCFTAHELMENQDVQQRLYEEVQQV
DQDLEGKELTYEAIMGMKYLDQVVNEVLRKWPAAIAVDRECNKDITFDVDGQKVEVKKGDVIWLPTCGFH
RDPKYFENPMKFDPERFSDENKESIQPFTYFPFGLGQRNCIGSRFALLEAKAVIYYLLKDYRFAPAKKSC
IPLELITSGFQLSPKGGFWIKLVQRN

>NP_651082.1 Cyp6d4 [Drosophila melanogaster]
MFSLILLAVTLLTLAWFYLKRHYEYWERRGFPFEKHSGIPFGCLDSVWRQEKSMGLAIYDVYVKSKERVL
GIYLLFRPAVLIRDADLARRVLAQDFASFHDRGVYVDEERDPLSANIFSLRGQSWRSMRHMLSPCFTSGK
LKSMFSTSEDIGDKMVAHLQKELPEEGFKEVDIKKVMQNYAIDIIASTIFGLDVNSFENPDNKFRKLVSL
ARANNRFNAMFGMMIFLVPSIAQFLFRIGFKNPVGLAMLQIVKETVEYREKHGIVRKDLLQLLIQLRNTG
KIDENDEKSFSIQKTPDGHIKTISLEAITAQAFIFYIAGQETTGSTAAFTIYELAQYPELLKRLQDEVDE
TLAKNDGKITYDSLNKMEFLDLCVQETIRKYPGLPILNRECTQDYTVPDTNHVIPKGTPVVISLYGIHHD
AEYFPDPETYDPERFSEESRNYNPTAFMPFGEGPRICIAQRMGRINSKLAIIKILQNFNVEVMSRSEIEF
ENSGIALIPKHGVRVRLSKRVPKLS

>NP_652020.1 Cyp4d14, isoform A [Drosophila melanogaster]
MYLELFAILLATALAWDYMRKRRHNKMYAEAGIRGPKSYPLVGNAPLLINESPKTIFDMQFRLIAEFGKN
IKTQMLGESGFMTADSKMIEAIMSSQQTIQKNNLYSLLVNWLGDGLLISQGKKWFRRRKIITPAFHFKIL
EDFVEVFDQQSATMVQKLYDRADGKTVINMFPVACLCAMDIIAETAMGVKINAQLQPQFTYVQSVTTASA
MLAERFMNPLQRLDFTMKLFYPKLLDKLNDAVKNMHDFTNSVITERRELLQKAIADGGDADAALLNDVGQ
KRRMALLDVLLKSTIDGAPLSNDDIREEVDTFMFEGHDTTTSSIAFTCYLLARHPEVQARVFQEVRDVIG
DDKSAPVTMKLLGELKYLECVIKESLRLFPSVPIIGRYISQDTVLDGKLIPADSNVIILIYHAQRDPDYF
PDPEKFIPDRFSMERKGEISPFAYTPFSAGPRNCIGQKFAMLEMKSTISKMVRHFELLPLGEEVQPVLNV
ILRSTTGINCGLKPRVY

>NP_610473.1 Cyp4p3 [Drosophila melanogaster]
MLILWLVGAFIVLIQWIYRLNRDYCILGFFAKRIRTKNGQNPESIAPLVKGSTIFANSFDLYGKDHSGVF

EHSRDCAKKLGKSYAEYAMGTAIYNVIDADSAERVLNDPNLINKGTIYDFLHPFLRTGLLTSTGKKWHAR
RKMLSPTFHFNILNQFQEIFITESLKFLEQFKGNDEAIISLNEVIPRFTLNSICETAMGVKLDEMAEKGD
RYRENFRQIEECFIRRMSNPLLWSDTLFKMFAEKDYASALDVVHGFSSEIIAKRRDQLNDEIDSRGNTQT
AEDELFTSKKRFAMLDTLILAEKDGLIDHIGICEEVDTLMFEGYDTTSIGLMFGLMNMSLYPEEQEKCYQ
EIQANIDDELNILNIGQLNKLKNLEYFIKETMRLFPSVPAMGRETTRETELSNGLILPKGSQIFVHVFDI
HRNPEYWDSPEEFRPERFLPENSQNRHTYAYIPFSAGQRNCIGQKFAMQEMKTLMVALLKQFQILPEIDP
KTIVFQTGLTLRTKNQIHVKLVRRK

>NP_609891.1 Cyp310a1 [Drosophila melanogaster]
MWLLLPILLYSAVFLSVRHIYSHWRRRGFPSEKAGITWSFLQKAYRREFRHVEAICEAYQSGKDRLLGIY
CFFRPVLLVRNVELAQTILQQSNGHFSELKWDYISGYRRFNLLEKLAPMFGTKRLSEMFGQVQKVGDHLI
HHLLDRQGQGCPQEVDIQQKLRVYSVNIIANLIYGLDINNFEHEDHILTSYLSHSQASIQSFTLGRLPQK
SSYTYRLRDLIKQSVELREDHGLIRKDILQLLVRFRNGNEVSGDKWQLEPINDADKLLSIKRLAKVAEDL
LKVSLDAVASTVTFTLLEILQEPLIVEKLRAEIKELSNENGQLKFEELNGLRYMDMCLKETLRKYPPLPI
IERVCRKSYSLPNSKFTIDEGKTLMVPLLAMHRDEKYFSEPMKYKPLRFLQTANDVGQCEDKTKSNVFIG
FGIGGSQCVGQNFAKLVIKVALIKLLQNFHLELDANQVKTLKVSHRPAPFIHTKDGLKVKLKRREINTKF
YS

>NP_609694.1 Cyp28a5 [Drosophila melanogaster]
MVLITLTLVSLVVGLLYAVLVWNYDYWRKRGVPGPKPKLLCGNYPNMFTMKRHAIYDLDDIYRQYKNKYD
AVGIFGSRSPQLLVINPALARRVFVSNFKNFHDNEIAKNIDEKTDFIFANNPFSLTGEKWKTRRADVTPG
LTMGRIKTVYPVTNKVCQKLTEWVEKQLRLGSKDGIDAKHMSLCFTTEMVTDCVLGLGAESFSDKPTPIM
SKINDLFNQPWTFVLFFILTSSFPSLSHLIKLRFVPVDVERFFVDLMGSAVETRRAQLAAGKQFERSDFL
DYILQLGEKRNLDNRQLLAYSMTFLLDGFETTATVLAHILLNLGRNKEAQNLLREEIRSHLQDGTIAFEK
LSDLPYLDACVQETIRLFPPGFMSNKLCTESIEIPNKEGPNFVVEKGTTVVVPHYCFMLDEEFFPNPQSF
QPERFLEPDAAKTFRERGVFMGFGDGPRVCIGMRFATVQIKAAIVELISKFNVKINDKTRKDNDYEPGQI
ITGLRGGIWLDLEKL

>NP_608916.1 Cyp4ac1 [Drosophila melanogaster]
MWIALLGIPILLAVLTLLLKHINKTYFILSLTKRVRTEDGSPLESKVAIMPGKTRFGNNLDILNFTPASV
FNFVRESTAKAKGQNYLWYFLYAPMYNVVRPEEAEEVFQSTKLITKNVVYELIRPFLGDGLLISTDHKWH
SRRKALTPAFHFNVLQSFLGIFKEECKKFLNVLEKNLDAELELNQVIPPFTLNNICETALGVKLDDMSEG
NEYRKAIHAIEEVLIQRVCNPLMYYNWYFFVYGDYRKHLQNLRIVHDFSSRIIERKRQQFQQKQLGEVDE
FGRKQRYAMLDTLLAAEADGQIDHQGICDEVNTFMFEGYDTTSTCLIFTLLMLALHEDVQKKCYEEVENL
PEDSDDISMFQFNKLVYLECVIKESLRMFPSVPFIGRQCVEETVVNGMVMPKDTQISIHIYDIMRDPRHF

PKPDLFQPDRFLPENTVNRHPFAYVPFSAGQRNCIGQKFAILEMKVLLAAVIRNFKLLPATQLEDLTFEN
GIVLRTQENIKVKLSKRVK

>NP_608911.1 Cyp28d2 [Drosophila melanogaster]
MCPVTTFLVLVLTLLVLVYVFLTWNFNYWRKRGIKTAPTWPFVGSFPSIFTRKRNIAYDIDDIYEKYKDT
DNMVGVFTTRVPQLLVMCPEYIHKIYATDFRSFHNNEWRNFVNKKTDMILGNNPFVLTGDEWKERRSEIM
PALSPNRVKAVYPVSQSVCKKFVEYIRRQQQMATSEGLDAMDLSLCYTTEVVSDCGLGVSAQSFTDTPTP
LLKMIKRVFNTSFEFIFYSVVTNLWQKVRKFYSVPFFNKETEVFFLDIIRRCITLRLEKPEQQRDDFLNY
MLQLQEKKGLHTDNILINTMTFILDGFETTALVLAHIMLMLGRNPEEQDKVRKEIGSADLTFDQMSELPH
LDACIYETLRLFSPQVAARKLVTEPFEFANKNGRTVHLKPGDVVTIPVKALHHDPQYYEDPLTFKPERFL
ESNGGGMKSYRDRGVYLAFGDGPRHCPGMRFALTQLKAALVEILRNFEIKVNPKTRSDNQIDDTFFMATL
KGGIYLDFKDL

>NP_608457.1 Cyp6t1 [Drosophila melanogaster]
MIAVFSLIAAALAVGSLVLLPVVLRGGCLLVVTIVWLWQILHFWHWRRLGVPFVPAAPFVGNVWNLLRGA
CCFGDQFRELYESKEAAGRAFVGIDVLHNHALLLRDPALIKRIMVEDFAQFSSRFETTDPTCDTMGSQNL
FFSKYETWRETHKIFAPFFAAGKVRNMYGLLENIGQKLEEHMEQKLSGRDSMELEVKQLCALFTTDIIAS
LAFGIEAHSLQNPEAEFRRMCIEVNDPRPKRLLHLFTMFFFPRLSHRVGTHLYSEEYERFMRKSMDYVLS
QRAESGENRHDLIDIFLQLKRTEPAESIIHRPDFFAAQAAFLLLAGFDTSSSTITFALYELAKNTTIQDR
LRTELRAALQSSQDRQLSCDTVTGLVYLRQVVDEVLRLYPPTAFLDRCCNSRTGYDLSPWNGGSPFKLRA
GTPVYISVLGIHRDAQYWPNPEVFDPERFSAEQRQQHHPMTYLPFGAGPRGCIGTLLGQLEIKVGLLHIL
NHFRVEVCERTLPEMRFDPKAFVLTAHNGTYLRFVKNSL

>NP_572721.1 Cyp4g15, isoform A [Drosophila melanogaster]
MEVLKKDAALGSPSSVFYFLLLPTLVLWYIYWRLSRAHLYRLAGRLPGPRGLPIVGHLFDVIGPASSVFR
TVIRKSAPFEHIAKMWIGPKLVVFIYDPRDVELLLSSHVYIDKASEYKFFKPWLGDGLLISTGQKWRSHR
KLIAPTFHLNVLKSFIELFNENSRNVVRKLRAEDGRTFDCHDYMSEATVEILLETAMGVSKKTQDKSGFE
YAMAVMRMCDILHARHRSIFLRNEFVFTLTRYYKEQGRLLNIIHGLTTKVIRSKKAAFEQGTRGSLAQCE
LKAAALEREREQNGGVDQTPSTAGSDEKDREKDKEKASPVAGLSYGQSAGLKDDLDVEDNDIGEKKRLAF
LDLMLESAQNGALITDTEIKEQVDTIMFEGHDTTAAGSSFFLSLMGIHQDIQDRVLAELDSIFGDSQRPA
TFQDTLEMKYLERCLMETLRMYPPVPLIARELQEDLKLNSGNYVIPRGATVTVATVLLHRNPKVYANPNV
FDPDNFLPERQANRHYYAFVPFSAGPRSCVGRKYAMLKLKILLSTILRNYRVYSDLTESDFKLQADIILK
REEGFRVRLQPRTS

>NP_524771.1 cytochrome P450-4e1 [Drosophila melanogaster]

MWIVLCAFLALPLFLVTYFELGLLRRKRMLNKFQGPSMLPLVGNAHQMGNTPTEILNRFFGWWHEYGKDN
FRYWIGYYSNIMVTNPKYMEFILSSQTLISKSDVYDLTHPWLGLGLLTSTGSKWHKHRKMITPAFHFNIL
QDFHEVMNENSTKFIDQLKKVADGGNIFDFQEEAHYLTLDVICDTAMGVSINAMENRSSSVVQAFKDITY
TIKMRAFSPWKRNKYLFHFAPEYPEYSKTLKTLQDFTNEIIAKRIEVRKSGLEVGIKADEFSRKKMAFLD
TLLSSKVDGRPLTSQELYEEVSTFMFEGHDTTTSGVGFAVYLLSRHPDEQEKLFNEQCDVMGASGLGRDA
TFQEISTMKHLDLFIKEAQRLYPSVPFIGRFTEKDYVIDGDIVPKGTTLNLGLLMLGYNDRVFKDPHKFQ
PERFDREKPGPFEYVPFSAGPRNCIGQKFALLEIKTVVSKIIRNFEVLPALDELVSKDGYISTTLGLQPA
EKKSRDAHNHKYDPILSASMTLKSENGLHLRMKQRLVCDST

>NP_523850.1 cytochrome P450-9c1 [Drosophila melanogaster]
MVFVELSIFVAFIGLLLYKWSVYTFGYFSKRGVAHEKPIPLLGNIPWSVLMGKESYIKHSIDLHLRLKQH
KVYGVFNLRDPLYYLSDPELIRQVGIKNFDTFTNHRKGITEGFNDTSVISKSLLSLRDRRWKQMRSTLTP
TFTSLKIRQMFELIHFCNVEAVDFVQRQLDAGTSELELKDFFTRYTNDVIATAAFGIQVNSFKDPNNEFF
SIGQRISEFTFWGGLKVMLYILMPKLMKALRVPVMDMNNVDYFKKLVFGAMKYRKEQSIVRPDMIHLLME
AQRQFKAEQEGSAESAAQQDKAEFNDDDLLAQCLLFFSAGFETVATCLSFTSYELMMNPEVQEKLLAEIL
AVKEQLGEKPLDYDTLMGMKYLNCVVSESLRKWPPAFIVDRMCGSDFQLKDEEGEVVVNLREDDLVHINV
GALHHDPDNFPEPEQFRPERFDEEHKHEIRQFTYLPFGVGQRSCIGNRLALMEVKSLIFQLVLRYHLKPT
DRTPADMMSSISGFRLLPRELFWCKLESRGPA

>NP_523645.1 cytochrome P450-9b1 [Drosophila melanogaster]
MSFVEICLVLATIGLLLFKWSTGTFKAFEGRNLYFEKPYPFLGNMAASALQKASFQKQISEFYNRTRHHK
LVGLFNLRTPMIQINDPQLIKKICVKDFDHFPNHQTLNIPNERLVNDMLNVMRDQHWRNMRSVLTPVFTS
AKMRNMFTLMNESFAQCLEHLKSSQPIAAGENAFELDMKVLCNKLSNDVIATTAFGLKVNSFDDPENEFH
TIGKTLAFSRGLPFLKFMMCLLAPKVFNFFKLTIFDSTNVEYFVRLVVDAMQYREKHNITRPDMIQLLME
AKKESKDNWTDDEIVAQCFIFFFAAFENNSNLICTTAYELLRNLDIQERLYEEVKETQEALKGAPLTYDA
AQEMTYMDMVISESLRKWTLSAAADRLCAKDYTLTDDEGTKLFEFKAGDNINIPICGLHWDERFFPQPQR
FDPERFSERRKKDLIPYTYLPFGVGPRSCIGNRYAVMQAKGMLYNLMLNYKIEASPRTTRDMWESARGFN
IIPTTGFWMQLVSRK

>NP_523527.1 cytochrome P450-4e3 [Drosophila melanogaster]
MWLAVLALLVLPLITLVYFERKASQRRQLLKEFNGPTPVPILGNANRIGKNPAEILSTFFDWWYDYGKDN
FLFWIGYSSHIVMTNPKQLEYILNSQQLIQKSTIYDLLHPWLGHGLLTSFGSKWHKHRKMITPSFHFNIL
QDFHEVMNENSAKFMTQLKKASAGDTIIDFQEHANYLTLDVICDTAMGVPINAMEQRDSSIVQAFRDMCY
NINMRAFHPFKRSNRVFSLTPEFSAYQKTLKTLQDFTYDIIEKRVYALQNGGSKEDHDPSLPRKKMAFLD
TLLSSTIDGRPLTRQEIYEEVSTFMFEGHDTTTSGVSFSVYLLSRHPDVQRKLYREQCEVMGHDMNRSVS

FQEIAKMKYLDLFIKEAQRVYPSVPFIGRYCDKDYDINGSIVPKGTTLNLALILLGYNDRIFKDPHHFRP

ERFEEEKPAPFEYLPFSAGPRNCIGQKFALLELKTVISKVVRSFEVLPAVDELVSTDGRLNTYLGLAPDE

KLKREAGRHKYDPILSAVLTLKSDNGLHLRLRERRS

AChE (17)

>P07140.1 RecName: Full=Acetylcholinesterase; Short=AChE; Contains: RecName: Full=Acetylcholinesterase 16 kDa subunit; Contains: RecName: Full=Acetylcholinesterase 55 kDa subunit; Flags: Precursor

MAISCRQSRVLPMSLPLPLTIPLPLVLVLSLHLSGVCGVIDRLVVQTSSGPVRGRSVTVQGREVHVYTGI

PYAKPPVEDLRFRKPVPAEPWHGVLDATGLSATCVQERYEYFPGFSGEEIWNPNTNVSEDCLYINVWAPA

KARLRHGRGANGGEHPNGKQADTDHLIHNGNPQNTTNGLPILIWIYGGGFMTGSATLDIYNADIMAAVGN

VIVASFQYRVGAFGFLHLAPEMPSEFAEEAPGNVGLWDQALAIRWLKDNAHAFGGNPEWMTLFGESAGSS

SVNAQLMSPVTRGLVKRGMMQSGTMNAPWSHMTSEKAVEIGKALINDCNCNASMLKTNPAHVMSCMRSVD

AKTISVQQWNSYSGILSFPSAPTIDGAFLPADPMTLMKTADLKDYDILMGNVRDEGTYFLLYDFIDYFDK

DDATALPRDKYLEIMNNIFGKATQAEREAIIFQYTSWEGNPGYQNQQQIGRAVGDHFFTCPTNEYAQALA

ERGASVHYYYFTHRTSTSLWGEWMGVLHGDEIEYFFGQPLNNSLQYRPVERELGKRMLSAVIEFAKTGNP

AQDGEEWPNFSKEDPVYYIFSTDDKIEKLARGPLAARCSFWNDYLPKVRSWAGTCDGDSGSASISPRLQL

LGIAALIYICAALRTKRVF

>AAM94376.1 acetylcholinesterase [Aphis gossypii]

MDQWLLWFGYLVASTYGLSLRHARHQSVGTPTAEEILEPQILIEDTDHVFRQRASDMFAQEPEYTEKRNL

NHRRRSEFSGNQDTNFESSGATYSAYTSDDPLIIHTNKGKIRGITQEATTGKLVDAWLGIPYAKKPIGDL

RFRHPRPIDRWDTTSPETILNCTTPPNTCVQIFDTLFGDFPGATMWNPNSPVSEDCLYINVVVPRPRPQN

AAVMVWIFGGGFYSGSATLDIYDPKILVSEENVILVSMQYRVASLGFLYFDTEDVPGNAGLFDQLMALQW

VHENIKLFGGNPNNVTLFGESAGAVSVSLHLLSPLSRNLFNQAIMESGSSTVPWAILSREESFSRGLKLA

KAMGCPDDRNEIHKTVECLRKVNSSAMVEKEWDHVAMCFFPFVPVVDGAFLDDHPQKSLSTNNFKKTNIL

MGSNSEEGYYFIFYYLTELFKKEENVVVSRENFIKAIGQLNPNADAAVKSAIEFEYTDWFNPNDPEKNRN

ALDKMVGDYQFTCNVNEFAHKYALTGNNVYMYYFKHRSLNNPWPKWTGVMHGDEISYVFGDPLNPNKRYE

IEEIELSKKMMRYWTNFAKTGNPSKTLEGSWVTPKWPVRTAYGKEFLTLDTNNTSIGVGPRLEQCAFWKN

YVPDLTVISKSMKSDKNCTTISGGTKTNMIKLSVWTIVMTTAVLML

>AAM94375.1 acetylcholinesterase [Aphis gossypii]

MSVDCVYTSAVTLLLCCSAVLGRPSSNGGADAGGGGGGGGGAGGAGGGGAGGGGGGGSAVDDTDEIPVVV

TSTGLVQGYTKIIANREVRVYTGIPFAKPPVGQLRFRRPVPVDPWTGVLNATRLPNTCYQERYEYFPGFV

GEEMWSPNTKLSEDCLYLNIWIPKKQRTRHHSNNAHHAKIPVLVWIYGGGYMSGTSTLDIYDGDLLAATF

DVMIASMQYRLGAFGSLYLTPELPEDSDDAPGNMGLWDQALAIKWIKENAAAFGADPETITLFGESAGGG
SVSVHLISPETGGMVKRGIIQSGTVNAPWSYMTGERAVEIAKKLLDDCNCNSTSLDSNPIGTMSCMRPVD
ASTISKKQWNSYSGILGFPSAPTVDGILLPEHPLDMLAKANFSDIDILIGSNLNEGTYFLLYDFVDFFDR
TSATALPREKFVQIVNVIFKDRTQLERDAIIYQYSGWEKKEVDDIYSNQKQLSDVVADYFFVCPTNLFAN
IVSSRGARVYYYFFTHRTDSHLWGDWMGVLHGDEMQYVFGHLLNMSMPYNARERDLSIRIMEAFTRFSLT
GTPVSDDIDWPLYNESKPIYHVWNAAEMHVGYGPRAAECQFWNGFFPKIAQALKETSKTTCEDYPDSMPT
INENCTFTSSFATVNPQISFTIIFIFVLPAYGLF

>AAL99585.1 acetylcholinesterase precursor [Myzus persicae]
MSVDCVYTSAVTLLLCCSAVLGRPSSNGGADGGGGGGGGGGAGAGGSGGGGGGGGGGSGVDDTDESPVVV
TSSGMVQGYTKIIANREVRVYTGIPFAKPPVGPLRFRRPVAVDPWTGVLNATRLPNTCYQERYEYFPGFV
GEEMWNPNTKLSEDCLYLNIWIPKKQRTRHHSNNAHHAKIPVLVWIYGGGYMSGTSTLDIYDGDLLAATF
DVMIASMQYRLGAFGSLYLTPELPEDSDDAPGNMGLWDQALAIKWIKENAAAFGADPETITLFGESAGGG
SVSVHLISPETRGMVRRGIIQSGTVNAPWSYMTGERAVDIAKKLLDDCNCNSTTLDSNPIATMSCMRAVD
ASTISKKQWNNYSGILGFPSAPTVDGVLLPEHPLDMLAKANFSDIDILIGSNLNEGTYFLLYDFVDFFDR
TSATALPKEKFVQIVNVIFKDKTQLERDAIIYQYSGWEKKDVDDKYSNQKQLSDVVADYFFVCPTNLFAN
IVSSRGARVYYYFFTHRTDSHLWGEWMGVLHGDEMQYVFGHPLNMSMPYNARERDLSIRIMEAFTRFSLT
GTPVSDDIDWPLYNESNPIYHVWNAAELHVGYGPRAAECQFWNGFFPKIAQALKETSKITCEDYPDSMPT
TNENCTFTSSFASINPQISFAIIFIFVLPAHGLL

>AAN71600.1 acetylcholinesterase 2, partial [Myzus persicae]
MDQWLLWFSYLVASTYGLSLRHARHQSVGTPTAEEILEPQILIEDTDHVFRQRALDMFAQEPEYTEKRNL
NHRRRSEFSGNQDNDFESSGETYSAYKSDDPLVIHTNKGKIRGITQAASTGKLVDAWLGIPYAKKPIGDL
RFRHPRPIDRWDNTNPETILNCTTPPNTCVQIFDTLFGDFPGATMWNPNSPVSEDCLYINVVVPKPRPQN
AAVMVWIFGGGFYSGSATLDIYDPKVLVSEENVILVSMQYRVASLGFLYFDTEDVPGNAGLFDQLMALQW
VHENIKLFGGNPNNVTLFGESAGAVSVSLHLLSPLSRNLFNQAIMESGSSTAPWAILSREESYSRGLRLA
RAMGCPDDRNEIHKTVECLRKANSSTMVEKEWDHVAICFFPFVPVVDGAFLDDYPQKSLSTNNFKKTNIL
MGSNSEEGYYSIFYYLTELFKKEENVVVSRENFVKAIGQLNPNADAAVKSAIEFEYTDWFSPNDPEKNRN
ALDKMVGDYQFTCNVNEFAHKYALTGNNVYMYYFKHRSLNNPWPKWTGVMHGDEISYVFGDPLNPNKRYE
IEEIELSKKMMRYWTNFAKTGNPSKTLKSWVTRQWPVHTAYGKEFLTLDTNNTSIACKTRQCA

>Q9TX11 Acetylcholinesterase, ACE (Acetylcholinesterase)
MKMSAVVRLCCNMISLLLCITVISPVYGIFDRLVVQTSSGPIRGRSTMVLGREVHVFNGVPFAKPPVDGL
RFRKPVPAEPWHGVLDATRLPPSCIQERYEYFPGFAGEEMWNPNTNVSEDCLYLNIWVPTKTRLRHGRGL
NFGNNDYFQDDDDFQRQHQSKGGLAMLVWIYGGGFMSGTSTLDVYNAEMLAAVGNVIVASMQYRVGSFGF

FYLAPYLNDDDAPGNVGLWDQALAIRWLKENAKAFGGDPDLITLFGESAGGSSVSLHLLSPVTRGLSRRG

ILQSGTLNAPWSHMSAEKALSVAEALIDDCNCNVTLLKDNPNYVMNCMRNVDAKTISVQQWNSYSGILGF

PSAPTIDGVFMTADPMTMLREANLEGVEILVGSNRDEGTYFLLYDFIDYFEKDAATSLPRDKFLEIMNTI

FSKASEPEREAIIFQYTGWESGNDGYQNQQQVGRSVGDHFFICPTNEFALGLAERGASVYYYYFTHRTST

SLWGEWMGVLHGDEVEYIFGQPMNVSMQYRQRERDLSRRMVLSVSEFARSGNPALEGEHWPVYTKENPIY

FIFNAEGEDDLRGEKYGRGPMATACAFWNDFLPRLRAWSVPPKSSCNILEQTSAATILYVDIKIVTVLMV

FILVRLY

>sp|Q869C3|ACES_ANOGA Acetylcholinesterase OS=Anopheles gambiae GN=Ace PE=3 SV=3

MEIRGLLMGRLRLGRRMVPLGLLGVTALLLILPPFALVQGRHHELNNGAAIGSHQLSAAA

GVGLASQSAQSGSLASGVMSSVPAAGASSSSSSSLLSSSAEDDVARITLSKDADAFFTPY

IGHGESVRIIDAELGTLEHVHSGATPRRRGLTRRESNSDANDNDPLVVNTDKGRIRGITV

DAPSGKKVDVWLGIPYAQPPVGPLRFRHPRPAEKWTGVLNTTTPPNSCVQIVDTVFGDFP

GATMWNPNTPLSEDCLYINVVAPRPRPKNAAVMLWIFGGGFYSGTATLDVYDHRALASEE

NVIVVSLQYRVASLGFLFLGTPEAPGNAGLFDQNLALRWVRDNIHRFGGDPSRVTLFGES

AGAVSVSLHLLSALSRDLFQRAILQSGSPTAPWALVSREEATLRALRLAEAVGCPHEPSK

LSDAVECLRGKDPHVLVNNEWGTLGICEFPFVPVVDGAFLDETPQRSLASGRFKKTEILT

GSNTEEGYYFIIYYLTELLRKEEGVTVTREEFLQAVRELNPYVNGAARQAIVFEYTDWTE

PDNPNSNRDALDKMVGDYHFTCNVNEFAQRYAEEGNNVYMYLYTHRSKGNPWPRWTGVMH

GDEINYVFGEPLNPTLGYTEDEKDFSRKIMRYWSNFAKTGNPNPNTASSEFPEWPKHTAH

GRHYLELGLNTSFVGRGPRLRQCAFWKKYLPQLVAATSNLPGPAPPSEPCESSAFFYRPD

LIVLLVSLLTATVRFIQ

>AAL33820.1 acetylcholinesterase [Plutella xylostella]

MIYNSKIVFTKLLLCICVCGARGRSWANHHDTTTSTTPAPTTTTPAPKNFHNDPLIVETKSGLVKGYAKT

VMGREVHIFTGIPFAKPPLGPLRFRKPVPIEPWHGVLEATAMPNSCYQERYEYFPGFEGEEMWNPNTNIS

EDCLYLNIWVPQHLRVRHHQDKPLTERPKVPILVWIYGGGYMSGTATLDLYKADIMASSSDVIVASMQYR

VGAFGFLYLNKYFSPGSEEAAGNMGLWDQQLAIRWIKDNARAFGGDPELITLFGESAGGGSVSLHMLSPE

MKGLFKRGILQSGTLNAPWSWMTGERAQDIGKVLVDDCNCNSSLLAVDPSLVMDCMRGVDAKTISVQQWN

SYTGILGFPSAPTVDGVFLPKDPDTMMKEGSFHNTEVLLGSNQDEGTYFLLYDFLDYFEKDGPSFLQREK

FLEIVDTIFKEFSKIKREAIVFQYTDWEEITDGYLNQKMIADIVGDYFFVCPTNYFAEVLADSGVDVYYY

YFTHRTSTSLWGEWMGVMHGDEMEYVFGHPLNMSLQYHTRERDLAAHIMQSFTRFALTGKPHKPEEKWPV

YSRASPHYYTYSADAASGPAPPRGPRASACAFWNHFLNKLNELEHVPCDGAVTGPYSSMAGKSLPIVLLT

ALATTAAL

>AAN37403.1 acetylcholinesterase [Helicoverpa armigera]

MISNTKIVFTKLLLCCFVSGAVARSWANHHDTTTSTTQTTPTTSPVPKNFHNDPLIVETKSGLVKGYAKT

VMGREVHIFTGIPFAKPPLGPLRFRKPVPIDPWHGVLEATAMPNSCYQERYEYFPGFEGEEMWNPNTNIS

EDCLYLNIWVPQHLRVRHHQDKPLAERPKVPILVWIYGGGYMSGTATLDLYKADIMASSSDVIVASMQYR

VGAFGFLYLNKYFSPGSEEAPGNMGLWDQQLAIRWIKDNARAFGGDPELITLFGESAGGGSVSLHMLSPE

MKGLFKRGILQSGTLNAPWSWMTGERAQDIGKVLVDDCNCNSSLLAADPSLVMDCMRGVDAKTISVQQWN

SYTGILGFPSAPTVDGVFLPKDPDTMMKEGNFHNTEVLLGSNQDEGTYFLLYDFLDYFEKDGPSFLQREK

FLEIVDTIFKDFSKIKREAIVFQYTDWEEITDGYLNQKMIADVVGDYFFVCPTNYFAEVLADSGVDVYYY

YFTHRTSTSLWGEWMGVMHGDEMEYVFGHPLNMSLQYHTRERDLAAHIMQSFTRFALTGKPHKPDEKWPL

YSRTSPHYYTYTADGTSGPAGPRGPRASACAFWNDFLNKLNELEHMPCDGAVTGPYSSVAGTTLPIVLLT

TLATTVAL

>AAM69920.1 acetylcholinesterase [Bactrocera oleae]

MAHPTSLLAGASLAAASSLSSRQYSVASSSRLSGDIGRGLFAIVVLLLRMSSVYGVIDRLVVQTSSGPVR

GRSVTVQGREVHVYTGIPYAKPPLDDLRFRKPVPAEPWHGVLDATRLPATCVQERYEYFPGFSGEEIWNP

NTNVSEDCLYINVWAPAKARLRHGRGANGGEHSNKADTDHLIHNGNPQNITNGLPVLIWIYGGGFMTGTA

TLDIYNADIMSAVGNVIVASFQYRVGAFGFLHLSPAMPGYEEEAPGNVGLWDQALAIRWLKTNAHAFGGN

PEWMTLFGESAGSSSVNAQLVSPVTAGLVKRGMMQSGTMNAPWSHMTSEKAVEIGKALINDCNCNASLLS

ENPQAVMACMRAVDAKTISVQQWNSYSGILSFPSAPTIDGAFLPDHPMKMMETADLRGYDILMGNVRDEG

TYFLLYDFIDYFDKDEATSLPRDKYLEIMNNIFGKVKPAERDAIIFRHTSWVGNPGLENQQQIGRAVGDH

FFTCPTNEYAQALAERGASVHYYYFTHRTSTSLWGEWMGVLHGDEIEYFFGQPSNTSLQYRQVERELGKR

MLNAVIEFAKTGNPATDGEEWPYFSKKDPVYYVFSTDDKEEKLQRGPLEGRCAFWNEYLREVRKWGSQCE

VKPSSASSLQQQQQHLLLQQRSIVAFMLALSLVLKIPSVNAFF

>AAM69372.1 acetylcholinesterase precursor [Musca domestica]

MARSVRTPTSPSSSSSSWSSWSSPSSSYFYSLLSSFKASLTRPSSSSSVAHHLAARNNDICRGLFATLVI

LLRMSALTSAMTDHLTVQTTSGPVRGRSVTVQGRDVHVFTGIPYAKPPVDDLRFRKPVPAEPWHGVLDAT

RLPATCVQERYEYFPGFSGEEMWNPNTNVSEDCLFMNIWAPAKARLRHGRGTNGGEHSSKTDQDHLIHSA

TPQNTTNGLPILIWIYGGGFMTGSATLDIYNAEIMSAVGNVIVASFQYRLGAFGFLHLSPVMPGFEEEAP

GNVGLWDQALALRWLKENARAFGGNPEWMTLFGESAGSSSVNAQLMSPVTRGLVKRGMMQSATMNAPWSH

MTSEKAVEIGKALVNDCNCNASLLPENPQAVMACMRQVDAKTISVQQWNSYSGILSYPSAPTIDGAFLPA

DPMTLLKTADLSGYDILIGNVKDEGTYFLLYDFIDYFDKDDATSLPRDKYLEIMNNIFQKASQAEREAII

FQYTSWEGNPGYQNQQQIGRAVGDHFFTCPTNEYAQALAERGASVHYYYFTHRTSTSLWGEWMGVLHGDE

IEYFFGQPLNNSLQYRPVERELGKRMLNSVIEFAKSGNPAVDGEEWPNFSKEDPVYYVFSTDEKIEKLQR

GPLAKRCSFWNDYLPKVRSWIGSECENKSSTSASAAIYEMKMQQLTLLAVAIILTMVNSIFQ

>AAK09373.1 acetylcholinesterase precursor [Schizaphis graminum]

MDQWLLWFGYLVASTYGLSLRHARHQSVGTPTAEEILEPQILIEDTDHVFRQRASDMFAQEPEYTEKRNL
NHRRRSEFSGNQDTDFASSGETYSAYTSDDPLIIHTNKGKIRGITQTATTGKLVDAWLGIPYAKKPIGDL
RFRHPRPIDRWDTTTPETILNCTTPPNTCVQIFDTLFGDFPGATMWNPNSPVSEDCLYINVVVPKPRPQN
AAVMVWIFGGGFYSGSATLDIYDPKILVSEENVILVSMQYRVASLGFLYFDTEDVPGNAGLFDQLMALQW
VHENIKLFGGNPNNVTLFGESAGAVSVSLHLLSPLSRNLFNQAIMESGSSTAPWAILSREESFNRGLKLA
KAMGCPDDRNTIHKTVECLRKANSSVMVEKEWDHVAICFFPFVPVVDGAFLDDHPQKSLSTNNFKKTNIL
MGSNSEEGYYSIFYYLTELFKKEENVMVSRENFIKAIGQLNPNADAAVKSAIEFEYTDWFSPNDPEKNRN
ALDKMVGDYQFTCNVNEFAHKYALTGNNVYMYYFKHRSLNNPWPKWTGVMHGDEISYVFGDPLNPNKRYE
IEEIELSKKMMRYWTNFAKTGNPSKTLEGSWVTPKWPVHTAYGKEFLTLDTNNTSIGVGPRLEQCAFWKN
YVPDLTAISKSMKSDKNCTTISGGTKTNVIELSVWTIVMTTAVLML

>Q27677.1 RecName: Full=Acetylcholinesterase; Short=AChE; Flags: Precursor

MGQLSILCLFVTVCASVCGYSWPSDETTTKPSQFKDFHTDPLVVETTSGLVRGYSKTVLGREVHVFTGIP
FAKPPIEQLRFKKPVPIDPWHGILDATKQPNSCFQERYEYFPGFEGEEMWNPNTNISEDCLYLNIWVPQR
LRIRHHADKPTIDRPKVPVLIWIYGGGYMSGTATLDVYDADIIAATSDVIVASMQYRLGSFGFLYLNRYF
PRGSDETPGNMGLWDQILAIRWIKDNAAAFGGDPDLITLFGESAGGGSISIHLISPVTKGLVRRGIMQSG
TMNAPWSYMSGERAEQIGKILIQDCGCNVSLLENSPRKVMDCMRAVDAKTISLQQWNSYSGILGFPSTPT
IEGVLLPKHPMDMLAEGDYEDMEILLGSNHDEGTYFLLYDFIDFFEKDGPSFLQREKYHDIIDTIFKNMS
RLERDAIVFQYTNWEHVHDGYLNQKMIGDVVGDYFFVCPTNNFAEVAADRGMKVFYYYFTHRTSTSLWGE
WMGVIHGDEVEYVFGHPLNMSLQFNSRERELSLKIMQAFARFATTGKPVTDDVNWPLYTKDQPQYFIFNA
DKNGIGKGPRATACAFWNDFLPKLRDNSGSEEAPCVNTYLSKIRSSSNELLPPSTSLVLIWIMTLLNAL

>P56161.1 RecName: Full=Acetylcholinesterase; Short=AChE; Flags: Precursor

MFVNQRTRRPYMSVFVLVLGAAVICPAYGIIDRLVVQTSSGPIRGRSTMVQGREVHVFNGVPFAKPPVDS
LRFKKPVPAEPWHGVLDATRLPPSCIQERYEYFPGFAGEEMWNPNTNVSEDCLYLNIWVPTKTRLRHGRG
LNFGSNDYFQDDDDFQRQHQSKGGLAMLVWIYGGGFMSGTSTLDIYNAEILAAVGNVIVASMQYRVGAFG
FLYLAPYINGYEEDAPGNMGMWDQALAIRWLKENAKAFGGDPDLITLFGESAGGSSVSLHLLSPVTRGLS
KRGILQSGTLNAPWSHMTAEKALQIAEGLIDDCNCNLTMLKESPSTVMQCMRNVDAKTISVQQWNSYSGI
LGFPSAPTIDGVFMTADPMTMLREANLEGIDILVGSNRDEGTYFLLYDFIDYFEKDAATSLPRDKFLEIM
NTIFNKASEPEREAIIFQYTGWESGNDGYQNQHQVGRAVGDHFFICPTNEFALGLTERGASVHYYYFTHR
TSTSLWGEWMGVLHGDEVEYIFGQPMNASLQYRQRERDLSRRMVLSVSEFARTGNPALEGEHWPLYTREN
PIFFIFNAEGEDDLRGEKYGRGPMATSCAFWNDFLPRLRAWSVPSKSPCNLLEQMSIASVSSTMPIVVMV
VLVLIPLCAWWWAIKKNKTPPHPQVILETRAFMH

>AAG43568.1 acetylcholinesterase, partial [Apis mellifera]

MTTRILLLFFLLLSSCTRPSRGNAVPSSQRGNVHNDPLVVETTSGLVRGFPRTVLDKEVHVFYGIPFAKP
PIGPLRFRKPLPIEPWHGVLNATVLPNSCYQERYEYFPGFPGEEMWNPNTNISEDCLYLNIWVPQKYRLR
HKGDGSPGGNGGPRNGLLPLLVWIYGGGFMSGTATLDVYNADIMAATSNVIIASMQYRVGAFGFLYLNKH
FTNSEEAPGNMGLWDQALALRWLRDNAEAFGGDPELITIFGESAGGSSVSLHLISPVTRGLVRRGILQSG
TLNAPWSYMSGEKANEVATILVDDCGCNSTMLNENPARVMACMRSVDAKTISVQQWNSYWGILGFPSAPT
IDGIFLPKHPLDLLREADFKDTEILIGNNENEGTYFILYDFNDIFEKDQASFLERERFLGIINNIFKNMS
QIEREAITFQYTDWEEVYNGYIYQKMVADVVGDYFFICPSIHFAQLFADRGMKVYYYFFTQRTSTNAMGR
VD

>AAC02779.1 acetylcholinesterase [Lucilia cuprina]

MARFITTSSSPTLTTSTAATAPSSSWSSNATSTATSISSHSRTSRKSRYTSSNLLNAFASLTSRSSLSLS
STSSNDLYRGFLTTLVILLRMSSVAYGITDRLIVQTTSGPVRGRAVTVQGREVHVFTGIPYAKPPVDDLR
FRKPVPAEPWHGVLDATRLPATCVQERYEYFPGFSGEEIWNPNTNVSEDCLYMNIWAPAKARLRHGRGAN
GGEHSSKTDPDHLIHSATPQNTTNGLPILIWIYGGGFMTGSATLDIYNADIMSAVGNVIVASFQYRVGAF
GFLHLSPVMPGFEEEAPGNVGLWDQALALRWLKENARAFGGNPEWMTLFGESAGSSSVNAQLVSPVTRGL
VKRGMMQSGTMNAPWSHMTSEKAVEIGKALINDCNCNASLLPANPQSVMACMRAVDAKTISVQQWNSYSG
ILSFPSAPTIDGAFLPADPMTLMKTADMSGYDIMIGNVKDEGTYFLLYDFIDYFDKDEATSLPRDKYLEI
MNNIFNKATQAEREAIIFQYTSWEGNPGYQNQQQIGRAVGDHFFTCPTNEYAQALAERGAQVHYYYFTHR
TSTSLWGEWMGVLHGDEIEYFFGQPLNTSLQYRAVERELGKRMLNSVIEFAKTGNPAVDGEEWPNFSKED
PVYYVFSTDEKTEKLQRGPLAKRCSFWNDYLPKVRSWVGSECENNSAESAAVSIIYEKQQNLLKWVIMLT
IMVTCIFQ

>AAF65202.1 acetylcholinesterase precursor [Nephotettix cincticeps]

MARLRFSTLSLSLLVAVATQPQPSTPRTLHSNDHNHGFLNEHKHSHAHAYKSHDRAHNTHAQFAEATGPA
STPSGGTPKHGDPLIVETTSGLVRGLSKTVLGREVHVFTGIPFAKPPVGPLRFRRPVPVDPWHGVYDATT
LSNSCYQERYEYFPGFEGEEMWNPNTNISEDCLYLNIWVPQRLRIRHKSSSEENTYRQKVPVLIWIYGGG
YMSGTATLDIYDADMVAATSDVIVASMQYRVGAFGFLYLSPELPPGSEEAPGNLGLWDQALAIQWIKANI
ANFGGDPELCTLFGESAGGGSVSLHLVSPVTRGLVRRGIMQSGTLNAPWSYMTGERAVEIAKTLIDDCGC
NASMLIESPSRVMSCMRAVDAKTISVQQWNSYFGILGFPSAPTIDGVFLPKHPLDLLKEGDFQDTEILIG
SNQDEGTYFILYDFIDYFEKDGPSFLQRDKFLDIINTIFKNFTRLERDAIIFQYTDWEHANDGYLNQKMI
GDVVGDYFFICPTNLFAQAFSDHGLKVFYYFFTQRTSTSLWGEWMGVMHGDEIEYVFGHPLNMSLQYNAR
ERDLSLRIMQAYSRFALTGKPVSDDINWPIYSREQPQYYIFNAEKSGIGKGPRATACAFWNEFLPRLRGQ
PDPECLADVAEVETSSPLVDNVSDNSTSTTFKPCTVITVLGLLLLTI

Rdl (3)

>XP_316071.4 AGAP006028-PA [Anopheles gambiae str. PEST]

MSLTIEVPHAKSPSLGVLILTLNLALFLPQTINRTPPYVLAGTGGGSMLGDVNISAILDSFSVGYDKRVR

PNYGGPPVEVGVTMYVLSISSLSEVKMDFTLDFYFRQFWTDPRLAYRKRPGVETLSVGSEFIKNIWVPDT

FFVNEKQSYFHIATTSNEFIRIHHSGSITRSIRLTITASCPMNLQYFPMDRQLCHIEIESFGYTMRDIRY

FWKDGLSSVGMSSEVELPQFRVLGHRQRATEINLTTGNYSRLACEIQFVRSMGYYLIQIYIPSGLIVIIS

WVSFWLNRNATPARVALGVTTVLTMTTLMSSTNAALPKISYVKSIDVYLGTCFVMVFASLLEYATVGYMA

KRIQMRKQRFMAIQKIAEQKKQQQADANHPPPPPPVASDHSHGHGHGHSHTHQHTPKQQMGSRSGTMQNV

PPNMGSRGCSIVGPLFQEVRFKVHDPKAHSKGGTLENTINGGRGGGQPGGGGPPPGGPPGGGGGGPDEES

AAPQHLIHPGKDINKLLGITPSDIDKYSRIVFPVCFVCFNLMYWIIYLHVSDVVADDLVLLGEEK

>NP_001261615.1 resistant to dieldrin, isoform E [Drosophila melanogaster]

MSDSKMDKLARMAPLPRTPLLTIWLAINMALIAQETGHKRIHTVQAATGGGSMLGDVNISAILDSFSVSY

DKRVRPNYGGPPVEVGVTMYVLSISSVSEVLMDFTLDFYFRQFWTDPRLAYRKRPGVETLSVGSEFIKNI

WVPDTFFVNEKQSYFHIATTSNEFIRVHHSGSITRSIRLTITASCPMNLQYFPMDRQLCHIEIESFGYTM

RDIRYKWNEGPNSVGVSSEVSLPQFKVLGHRQRAMEISLTTGNYSRLACEIQFVRSMGYYLIQIYIPSGL

IVIISWVSFWLNRNATPARVALGVTTVLTMTTLMSSTNAALPKISYVKSIDVYLGTCFVMVFASLLEYAT

VGYMAKRIQMRKQRFMAIQKIAEQKKQQLDGANQQQANPNPNANVGGPGGVGVGPGGPGGPGGGVNVGVG

MGMGPEHGHGHGHHAHSHGHPHAPKQTVSNRPIGFSNIQQNVGTRGCSIVGPLFQEVRFKVHDPKAHSKG

GTLENTVNGGRGGPQSHGPGPGQGGGPPGGGGGGGGGGPPEGGGDPEAAVPAHLLHPGKVKKDINKLLG

ITPSDIDKYSRIVFPVCFVCFNLMYWIIYLHVSDVVADDLVLLGEE

>AEB60992.1 GABA receptor [Anopheles funestus]

MSLTIEVPHAKSPSLGVLILTLNLALFLPQTINRTPPYVLAGTGGGSMLGDVNISAILDSFSVGYDKRVR

PNYGGPPVEVGVTMYVLSISSLSEVRMDFTLDFYFRQFWTDPRLAYRKRPGVETLSVGSEFIKNIWVPDT

FFVNEKQSYFHIATTSSEFIRIHHSGSITRSIRLTITASCPMGLQYFPMDRQLCHIEIESFGYTMRDIRY

KWNEGPNSVGVSSEVSLPQFKVLGHRQRAMEISLTTGNYSRLACEIQFVRSMGYYLIQIYIPSGLIVIIS

WVSFWLNRDATPARVSLGVTTVLTMTTLMSSTNAALPKISYVKSIDVYLGTCFVMVFASLLEYATVGYMA

KRIQMRKQRFMAIQKIAEQKKQQAADANHPPPPPPVASDHSHGHGHGHSHTHQHTPKQQMGSRSGTMQNV

PPNMGSRGCSIVGPLFQEVRFKVHDPKAHSKGGTLENTINGGRGGGQPGGGGGGGPPGGPPGGGGGGPDE

ESAAPQHLIHPGKDINKLLGITPSDIDKYSRIVFPVCFVCFNLMYWIIYLHVSDVVADDLVLLGEEK

<u>Kdr (2)</u>

>ALB75309.1 voltage gated sodium channel subunit 2 transcript variant 1 [Rhopalosiphum padi]

MSVYSSEELLDAGIIYRNKKEQLDVTIGDGMELLIRGEKNKKKKPPTSSSYNSFGMHQSSTDENYYLKDK

YEYDTRSTKSYGSHEHDPYDSESHRGSKRSLHNAEEKKDPSKEDVEINQNSGNDGIAAEEINGDEYKEQG

PVEMVEDVFEEEEYPEDCFPPNCYKKFPFLAGDDETPFWLGWGQLRLKTFQLIENKYFETAVITMILLSS

LALALEDVHLQHRPVLQDILYYMDRIFTVIFFLEMLIKWLALGFRNYFTNAWCWLDFIIVMVSLINFVAA

LLGASGIQAFKTMRTLRALRPLRAMARMQGMRVVVNALVQAIPSIFNVLLVCLIFWLIFAIMGVQLFAGK

YYKCVDPKDGKTLNHEIIPDKNVCIAEMYKWENSKMNFDHMGNAYLCLFQVATFNGWMEIMRDAVDSRDT

HGKQPIREINNYMYFYFVFFIIFGSFFTLNLFIGVIIDNFNEQKKKTGASLEMFMTEDQKKYYNAMKKMS

SKKPLKAIPRPRWRPQSIVFQTVTDKKFDMLIMLFIGFNMLTMTLDHYQQTKLFTDVLERLNQIFIAIFS

TECLLKIFALRYYYFKEPWNLFDFVVVILSLAGLVLSDLISKYFVSPTLLRVVRVAKVGRVLRLVKGAKG

IRTLLFALAMSLPALFNICLLLFLVMFIFAIFGMSFFMNVDSHGGLDEDYNFRTFGQSMILLFMLSTSSG

WDAVLDGITNEENCQKPNLEMGITGSCGSSAVGTAFLLSYLVINFLIVINMYIAVILENYSQATEDVQEG

LTDDDYDMYYEIWQHFDPNGTRYIRYDQLSDFLDILEPPLKIHKPNKYKIVSMDIPICKGDLIYCVDILD

ALTKDFFARKGNPIIETVAEIGEMQTRPEEAGYEPISSSLWRMREVYCAIIIQNSWRKYTAAAKQQTADD

ERSDGAASPDGRETAVLVESDGFVTKNGHKVVIHSRSSSKSSRLADV

>AJS11555.1 voltage gated sodium channel variant 2 [Rhopalosiphum padi]

MSIADTDSSFSSEEKSLFRPFTRESLRAIEQRIAEDHAKQKELEEKRAEGEVRYEDEDEDEGPQPDATLE

QGAPLPVRLVGTFPPELASVPLEDIDPYYHNQKTFVVISKGKDIFRFSATDGLWALDPFNPIRRVAIYIL

VHPIFSVTIITTILTNCVFMIMPPTPTIEASEVIFTGIYTFESAVKVMARGFILEHFTYLRDAWNWLDFI

VIALAYVTMGIELGNLAVLRTFRVLRALKTVAIVPGLKTIVGAVIESVKNLRDVIILTIFSLSVFALLGL

QIYMGVLTQKCIKYFPTDGSAGNLTNENWFAFMSNSSNWQPGEEEPDEYPLCGNGTGAGQCKEGYMCIQG

FGINPNYGYTSFDTFAWALLSAFRLMTQDNWEALYQQVLRAAGPWHMFFFIVIIFLGSFYLVNLILAIVA

MSYDELQKKAEEEEAAEEEAIREAEQAAKDREVRRQAHEERVAERAERARHVTQHPKSPSDFSCQSYDNM

FADGQDRGIGNDHHREKMSLRSVSITSHDKNSDTGSVDRQSGKTRKASLSLPGSPFNIRRASRGSHQLSH

RNGRPRFTGADTKPLVLNTLLDAEEHLPYADDSNAVTPMSEENGAIIVPVSYANFGSRHSSYTSHTSRIT

YTSHADLFKPPMTKERQLRSRSARNYFNPSDQRYHRDDDYDSSSMSKSKQKVDECGYNDSQKHTVVDMRD

VMVLNDIIEQAAGRQSRGSEKAESTVYVFPTDEDAVDGEDEEEEDEEPTFREKFQVWLLKFIDTFCVWDC

GWPWLKFQQGLAFIVFDPFVELYITLCIVVNTLFMALDHHEMDPKLDFILNKANVFFSATFGVEAALKLM

AMSPKYYFQMGWNIFDFIIVILSVVELLSAGYQGLSVLRSFRLLRVFKLAKSWPTLNLLISIMGRTIGAL

GNLTFVLCIIIFIFAVMGMQLFGKNYTEKMYLFRDHELPRWNFTDFLHSFMIVFRVLCGEWIESMWDCLH

VGEPTCIPFFLATVVIGNLVVLNLFLALLLSNFGSSNLSVPTADSDTNKITEAFERIGRFNKWMKTHIMN

FLKALRAKITNQISVQASGRDRDIDLPVDETIVDVIAPFKDTKEPVEMTIGDGMEFTIPGDVKQKIKKNQ

VGNSIGNHQGNKVGNDYKKESFDLDSLKC
